# Mapping ECoG channel contributions to trajectory and muscle activity prediction in human sensorimotor cortex

**DOI:** 10.1038/srep45486

**Published:** 2017-03-31

**Authors:** Yasuhiko Nakanishi, Takufumi Yanagisawa, Duk Shin, Hiroyuki Kambara, Natsue Yoshimura, Masataka Tanaka, Ryohei Fukuma, Haruhiko Kishima, Masayuki Hirata, Yasuharu Koike

**Affiliations:** 1 Institute of Innovative Research, Tokyo Institute of Technology, Yokohama, Japan; 2 Division of Clinical Neuroengineering, Global Center for Medical Engineering and Informatics, Osaka University, Osaka, Japan; 3 Department of Neurosurgery, Osaka University Medical School, Osaka, Japan; 4 ATR Computational Neuroscience Laboratories, Japan; 5 Division of Functional Diagnostic Science, Osaka University Graduate School of Medicine, Osaka, Japan; 6 Department of Electronics and Mechatronics, Tokyo Polytechnic University, Atsugi, Japan

## Abstract

Studies on brain-machine interface techniques have shown that electrocorticography (ECoG) is an effective modality for predicting limb trajectories and muscle activity in humans. Motor control studies have also identified distributions of “extrinsic-like” and “intrinsic-like” neurons in the premotor (PM) and primary motor (M1) cortices. Here, we investigated whether trajectories and muscle activity predicted from ECoG were obtained based on signals derived from extrinsic-like or intrinsic-like neurons. Three participants carried objects of three different masses along the same counterclockwise path on a table. Trajectories of the object and upper arm muscle activity were predicted using a sparse linear regression. Weight matrices for the predictors were then compared to determine if the ECoG channels contributed more information about trajectory or muscle activity. We found that channels over both PM and M1 contributed highly to trajectory prediction, while a channel over M1 was the highest contributor for muscle activity prediction.

Studies on brain-machine interface (BMI) have demonstrated success using electrocorticography (ECoG) to predict hand trajectory in monkeys^1^,^2^,^3^,^4^ and hand^5^,^6^,^7^,^8^ and finger^9^,^10^,^11^,^12^,^13^,^14^ trajectory in humans, decode joint angle^15^ and grasping force^16^ and estimate muscle activity^17^,^18^. Advances in ECoG electrodes have allowed for more precise prediction of human hand and finger movements^19^,^20^. Control of prosthetic limbs using ECoG has also been achieved^21^,^22^,^23^. Therefore, ECoG offers considerable potential in rehabilitation as a control modality for neuro-prostheses.

A topic of debate has arisen, however, on whether activity from the primary motor cortex (M1) and premotor cortex (PM) represents “extrinsic” parameters related to the location of a target, or “intrinsic” parameters related to a set of muscles^24^,^25^,^26^,^27^,^28^,^29^,^30^. For example, Kakei *et al*. showed that most neurons in the monkey ventral premotor cortex exhibited “extrinsic-like” encoding (~94%), while M1 neurons exhibited both “intrinsic-like” (~32%) and “extrinsic-like” (~61%) encoding^29^.

This disparity gives rise to further questions: When predicting hand and finger trajectories using ECoG, are the predictions based on extrinsic or intrinsic information? When estimating arm muscle activity, is only intrinsic information exploited? It is unlikely that ECoG activity can be divided into extrinsic and intrinsic components based on signal amplitude or other features, because the ECoG signal is a summation of signals from a huge number of neurons beneath an electrode. We expect that ECoG signals from PM and some parts of M1 would mainly result from firing of extrinsic-like neurons, while other signals from M1 would result from both extrinsic-like and intrinsic-like neurons firing.

Seeking answers to the above questions, here we performed trajectory and muscle activity predictions for object-carrying tasks designed to vary muscle activity but keep trajectory unchanged. We obtain decoders trained with ECoG signals of various object masses and compare spatial distributions of weight matrices for trajectory prediction with those for muscle activity prediction. If the predictor is constructed based on ECoG signals derived from both extrinsic-like and intrinsic-like neurons, predicted trajectories will stray from actual trajectories given objects of different mass. Conversely, if the weight matrix for muscle activity prediction is obtained based on signals derived from extrinsic-like neurons, the predicted muscle activity will not change with varying object mass. Similar results would be expected if trajectory were varied while muscle activities were kept constant. We also trained predictors using task data for a single object mass to observe the effects of object mass on prediction.

## Materials and Methods

### Ethical approval

The study protocol was approved by the ethics committee of Osaka University Hospital (Approval No. 14353, UNIM ID: UMIN000017900) and carried out in accordance with the Declaration of Helsinki. The ECoG electrodes in this study were implemented as part of the patients’ treatment. All patients or their guardians gave written informed consent before participating in the study.

### Participants and electrodes

**Figure 1:**
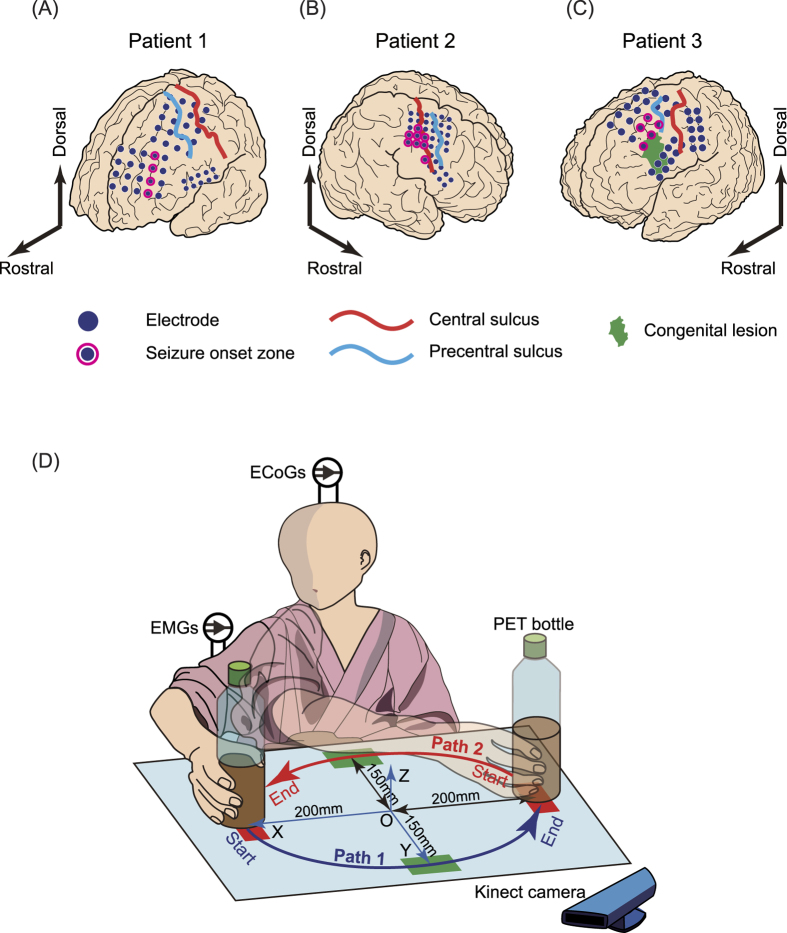
ECoG electrodes and behavioral tasks. (**A**–**C**) Dark blue circles denote platinum planar electrodes on the cerebral cortices. Red and cyan blue lines are the central and precentral sulcus, respectively. The electrodes were implanted on the left hemisphere of patients 1 and 3, and the right hemisphere of patient 2. Patient 3 had a cerebral congenital lesion (green area). The seizure onset zone also marked with red circles in the three patients. (**D**) All participants carried a bottle counterclockwise on a table. First, they placed it firmly on the start position (left red marker). Then they carried it along path 1 (blue arc) to the end position (right red marker) via a specified point (green marker). They placed the bottle firmly on the end point. They then carried the bottle from the along path 2 (red arc) to the end position (left red marker) and placed it firmly on the marker. They repeated the carrying task alternately for paths 1 and 2. ECoG and EMG signals were recorded with a 128-channel digital EEG system. Trajectories of a bottle cap were also recorded with the Kinect camera.

Three female patients aged 66, 11 and 23 years, hereinafter referred to as patients 1, 2 and 3, respectively, participated in this study. All patients were diagnosed with intractable epilepsy. The sensorimotor cortices of patients 1 and 2 were undamaged. Patient 3 had a cerebral congenital lesion on her right frontal lobe (green area in Fig. 1C). Subdural planar platinum electrode arrays (Unique Medical Co., Tokyo, Japan) covering the precentral and central sulcus were implanted in all patients to localize foci of epilepsy and functional mapping (Fig. 1A–C). Each electrode had an exposed surface diameter of 3 mm. Inter-electrode distance was 10 mm for patients 1 and 3, and 7 mm for patient 2. The electrodes were implanted for 9 days in patient 1 and 14 days in patients 2 and 3.

### Behavioral tasks

Patients 1, 2 and 3 conducted behavioral tasks 6 days, 13 days and 10 days after implantation, respectively. Sitting upright on their hospital room beds, the patients performed carrying tasks with a plastic bottle on a table (Fig. 1D). Patients 1 and 3 handled the bottle with their right arms, and patient 2 used her left arm. Four points were marked on the table with masking tape (red and green rectangles in Fig. 1D). Patients put the bottle at a start point (left red marker) and carried it to an end point (right red marker) via a green marker on a path denoted as path 1 (blue arc). The X and Y coordinates (mm) of the bottle changed from (200, 0) to (0, 150) and then from (0, 150) to (−200, 0). Carrying height for the bottle was not specified, but patients were required to place it firmly on the red markers for an instant. After a brief stop on the end point of path 1, they were to continue carrying along path 2 (red arc). The path 1 and 2 carrying tasks were alternately repeated about 20 times per session. Bottles with three different masses were prepared: an empty bottle (25 g), a half-full bottle (250 g) and a full bottle (500 g). Each patient performed three sessions, one with each of the three bottle masses.

### ECoG, EMG and motion recordings

Patient 1 was implanted with a 4 × 5 electrode array on the left cerebral hemisphere (Fig. 1A), patient 2 was implanted with a 5 × 6 and a 2 × 5 array on the right hemisphere (Fig. 1B) and patient 3 was implanted with a 4 × 5 array and two 2 × 5 arrays on the left hemisphere (Fig. 1C). A scalp electrode on the nasion of each patient was used as a reference for all electrodes. EMG signals of biceps and triceps brachii and anterior and posterior deltoid were recorded for all patients. ECoG and EMG were synchronously recorded with a 128-channel digital electroencephalography (EEG) system (Neurofax EEG 1200; Nihon Koden Corporation, Tokyo, Japan) at a sampling rate of 1000 Hz.

The 3D motion of the bottle was recorded with a Kinect for Windows sensor (Microsoft, Redmond, WA) at 30 fps, using the bottle’s cap as the reference point (Fig. 1D). Motion recording durations (average across three sessions ± standard deviation) and total number of trials across sessions with the 25-g, 250-g and 500-g bottles were as follows: 1.72 ± 0.17 s (40 trials), 1.53 ± 0.09 s (40 trials) and 1.52 ± 0.13 s (42 trials) for patient 1; 1.86 ± 0.29 s (51 trials), 1.99 ± 0.31 s (39 trials) and 1.91 ± 0.28 s (44 trials) for patient 2; and 2.97 ± 0.32 s (42 trials), 3.14 ± 0.4 s (40 trials) and 2.97 ± 0.33 s (42 trials) for patient 3; respectively. There were no significant differences in duration among weights (F_2,371_ = 0.25, p = 0.779, the two-way ANOVA with unweighted-means analysis).

To synchronize the Kinect images and ECoG/EMG signals, brief flashes of LED light were displayed at random intervals and recorded with the Kinect camera, while trigger signals of the flashes were recorded with the EEG system.

### ECoG and EMG signal processing

We applied the following ECoG pre-processing methods, which we previously proposed. (1) Raw ECoG signals were re-referenced to a common average reference. (2) The re-referenced signals were decomposed into nine frequency bands with fourth-order bandpass Butterworth filters (δ: ~4 Hz, θ: 4~8 Hz, α: 8~14 Hz, β1: 14~20 Hz, β2: 20~30 Hz, γ1: 30~60 Hz, γ2: 60~90 Hz, γ3: 90~120 Hz and γ4: 120~150 Hz). (3) These component signals were full-wave rectified. (4) They were smoothed with a Gaussian window (cutoff frequency: 3.98 Hz, time constant: 0.04 s). (5) The smoothed signals were down-sampled to 100 Hz. (6) Standard *z*-score *z*_*i*_(*t*) were obtained by normalization of the signals *x*_*i*_(*t*) (*i* = 1, 2, …. *n* × 9) at time *t*, such that


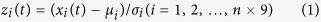


where *n*. μ_*i*_ and σ_*i*_ denote the number of electrodes, the mean value of *x*_*i*_(*t*) and the standard deviation of *x*_*i*_(*t*), respectively. We also used this method in our previous studies to predict trajectory^2^,^7^, muscle activation^17^, and force^18^, though not in our 3D motion study^14^, which used only unrectified ECoG. In the current study, we used both rectified and unrectified features in the weight selection.

EMG signals were band-pass filtered from 30 to 400 Hz and full-wave rectified. The signals were smoothed with a second-order low-pass filter with a cut-off frequency of 2.2 Hz^31^,^32^ and down-sampled from 1000 Hz to 100 Hz.

### Decoding method

The bottle position or muscle activity *Y*_*p*_(*t*) at a time *t* was predicted with sparse linear regression (SLiR)^32^,^33^,^34^,^35^ as follows.


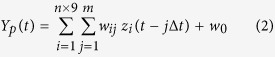


where *m* denotes the number of sampling points for the *z*-score used for prediction of *Y*_*p*_, and Δ*t* represents the time interval between sampling points. *w*_*0*_ and *w*_*ij*_ are a bias term and members belonging to a weight matrix. In this study, we assigned *m* = 100 points and Δ*t* = 0.01 s. We then performed a leave-one-out cross validation (LOO-CV) to verify our results. Finally, we obtained root-mean-square error (RMSE) and Pearson’s correlation coefficient (CC) by comparing predicted *Y*_*p*_ and actual values in LOO-CV.

## Results

### Actual bottle trajectories and muscle activity

**Figure 2:**
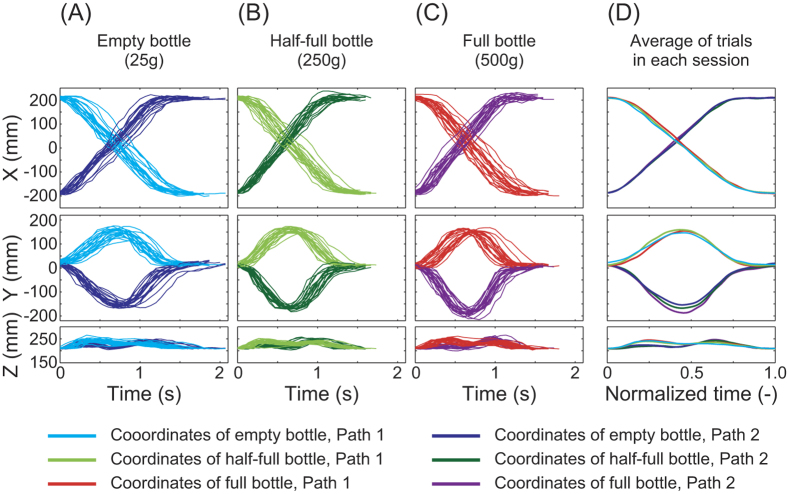
Actual trajectories for patient 1. (**A**–**C**) Coordinates using the 25-g, 250-g and 500-g bottles, respectively. (**D**) Coordinates averaged across trials in each session. The differences between average trajectories are small, especially in the X-direction.

Figure 2 shows actual bottle trajectories by patient 1 for all trials from the 25-g, 250-g and 500-g sessions (Fig. 2A–C) and averaged across trials after resampling each trial period to 200 points (Fig. 2D). Observed differences between sessions were slight, particularly in X coordinates. Actual trajectories for patients 2 and 3 are shown in Supplementary Figs S1 and S2, respectively. Supplementary Table S1 shows p and F values for a one-way ANOVA comparing coordinates for all trials and patients at 0.25 T, 0.5 T and 0.75 T, where T denotes each trial period. Patient 1 had the smallest differences among the three patients.

**Figure 3:**
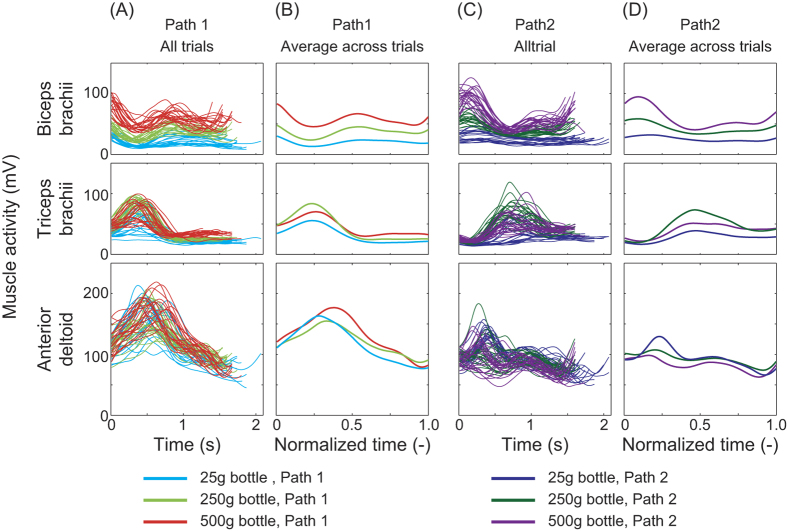
Actual biceps, triceps brachii and anterior deltoid activities of patient 1 for all trials (**A** and **C**) and averaged across all trials in each session (**B** and **D**). Biceps brachii activity showed more differences between sessions than the other two muscles.

Figure 3 shows actual biceps brachii, triceps brachii and anterior deltoid activity (pre-processed EMG signals) for all trials (Fig. 3A,C) and averaged across trials (Fig. 3B,D). Although EMG of the posterior deltoid was also measured, the signals were highly uniform across trials and sessions. Among the three muscles, the biceps brachii showed the largest difference in activity across sessions. The p and F values for a one-way ANOVA comparing actual muscle activity for all trials and patients are shown in Supplementary Table S2.

### Predicted bottle trajectories and muscle activity

**Figure 4:**
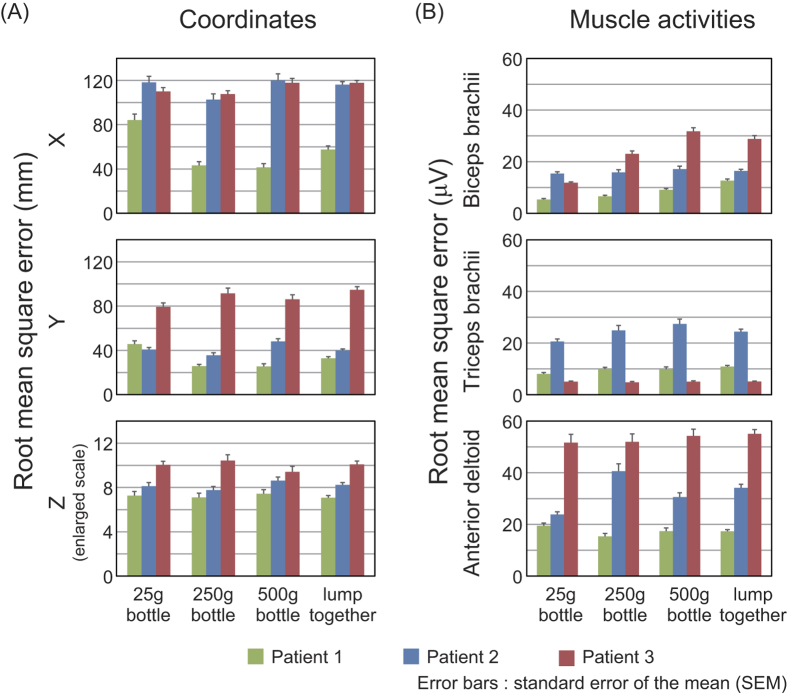
Root-mean-square errors (RMSE) of predicted compared to actual trajectories (**A**) and muscle activities (**B**). Data are shown for decoders trained with 25 g, 250 g and 500 g bottle masses, as well as for all three masses lumped together.

Bottle trajectories were predicted for all trials, sessions and patients using sparse linear regression and LOO-CV. Prediction results were evaluated with RMSE and CC. We trained decoders based on subsets of ECoG and coordinate data from each session, as well as from all three sessions lumped together, and used them to predict X, Y and Z coordinates. Figure 4A shows RMSE between predicted and actual trajectories. Note that the proposed method can also estimate the complete opposite direction with the same decoders even without all lumped data. Predictions for patient 1 showed relatively low RMSE values and were typically the best among the three patients. Patient 1 also showed higher CC values than the others (Supplementary Fig. S3A).

Muscle activity was also predicted in the same way as trajectory prediction. Patient 1 showed the lowest RMSE values for anterior deltoid activity as well as biceps brachii activity with the 250 g and 500 g bottles (Fig. 4B).

**Figure 5:**
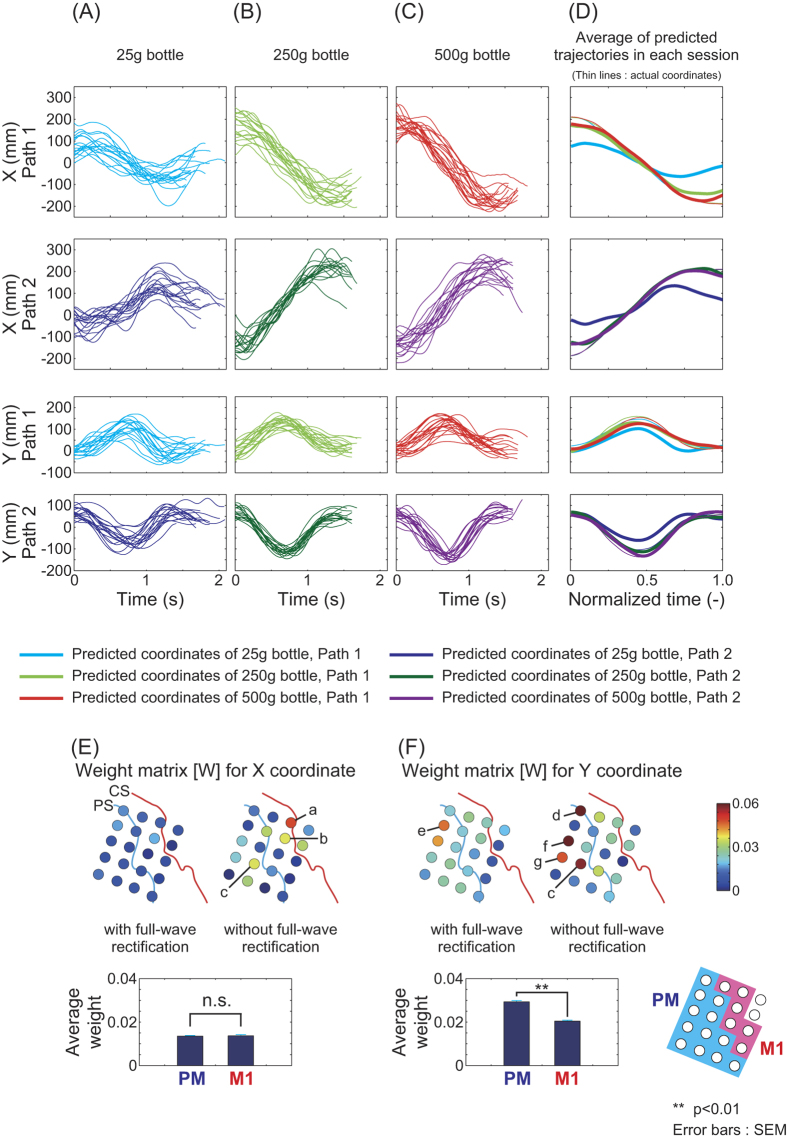
X and Y coordinates predicted using ECoG signals of patient 1. The decoders were trained with signals of all bottle masses and paths (“lump together” in Fig. 4A). One predictor was prepared for each coordinate axis. (**A**–**C**) Predicted coordinates for each trial using the 25-g, 250-g and 500-g bottles, respectively. (**D**) Averages across all trials in each session. Thin lines are actual trajectories. (**E**) Spatial distribution of the weight matrix for the X-coordinate predictor. Average weights for PM and M1 were not significantly different (p = 0.829, t-test). (**F**) Weight matrix for the Y-coordinate predictor. Average weight for PM was significantly higher than that for M1 (p = 1.15 × 10^−23^).

X- and Y-coordinate predictions for patient 1 are shown in Fig. 5. Average predicted trajectories (Fig. 5D, thick lines) for the 250 g and 500 g bottles fitted well with actual trajectories (thin lines). Figs. 5E and 5F show spatial distributions of normalized weight matrices |*w*_*ij*_ |/|*w*_*max*_| for the X- and Y-coordinate predictors, where |*w*_*max*_| denotes the maximum among absolute values of all members |*w*_*ij*_| in matrix [*W*]. For the X-coordinate predictor, channels labeled *a, b* and *c* for ECoG without full-wave rectification showed relatively high weight values. Weight averages for areas PM and M1 were not significantly different from each other (p = 0.829, t-test). Weight matrices for the Y-coordinate predictor showed relatively high values at channels *c* through *g*, and average weight for PM was significantly higher than that for M1 (p = 1.15 × 10^−23^).

**Figure 6:**
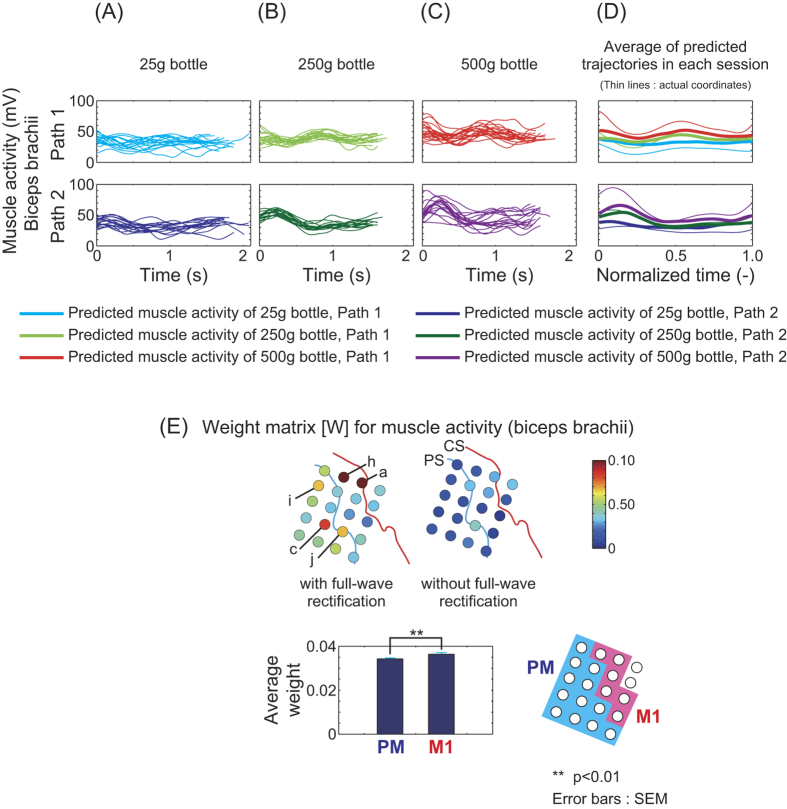
Biceps brachii activity predicted using ECoG signals of patient 1. Signals of all bottle masses and paths were used to train the predictor (“lump together” in Fig. 4B). (**A**–**C**) Predicted muscle activity of each trial using the 25-g, 250-g and 500-g bottles, respectively. (**D**) Averages across all trials in each session. Thin lines are actual muscle activities. (**E**) Spatial distribution of the weight matrix for the muscle activity predictor. Average weight for M1 was significantly higher than that for PM (p = 0.00916, t-test).

Figure 6 shows predicted biceps brachii activity for patient 1. Significant differences between predicted muscle activities were observed (thick lines in Fig. 6D). The p and F values for a one-way ANOVA comparing predicted muscle activity for all trials and patients are shown in Supplementary Table S3 (cf. Table S2). Muscle activity for patient 1 typically showed significant differences according to bottle weight.

Distributions for weight matrix [*W*] are shown in Fig. 6E. Channels *a, c, h, i* and *j* for ECoG with full-wave rectification had high weights. Average weight for M1 was significantly higher than that for PM (p = 0.00916). Predicted triceps brachii activity also yielded significantly higher average weights for M1 than PM, but those of the anterior deltoid did not (Supplementary Fig. S4). Though it is not clear whether M1 had more information than PM, the highest contributor for muscle activity prediction was channel *a* in M1. This could mean that intrinsic-like neurons for generating movement are more prominent than extrinsic-like neurons under the area of channel *a*.

Weights and weight ratios of the frequency bands for XY coordinates and biceps brachii activity predictors are shown in Supplementary Fig. S5. Weight values using full-wave rectified ECoG were higher at all frequency bands for the muscle activity predictor than the trajectory predictor. This shows that the higher frequency bands had slightly more influence on muscle activity prediction than trajectory prediction. The δ weights using unrectified ECoG and the δ and θ weights using rectified ECoG were higher than any other band. The ratio of δ frequency bands (dark blue and blue) notably favored XY coordinates, while ratios for θ and γ bands favored muscle activity. Particularly the γ bands, such as those for channels *a* and *h*, were greater in the M1 area.

### Effect of bottle mass on trajectory and muscle activity predictions

**Figure 7:**
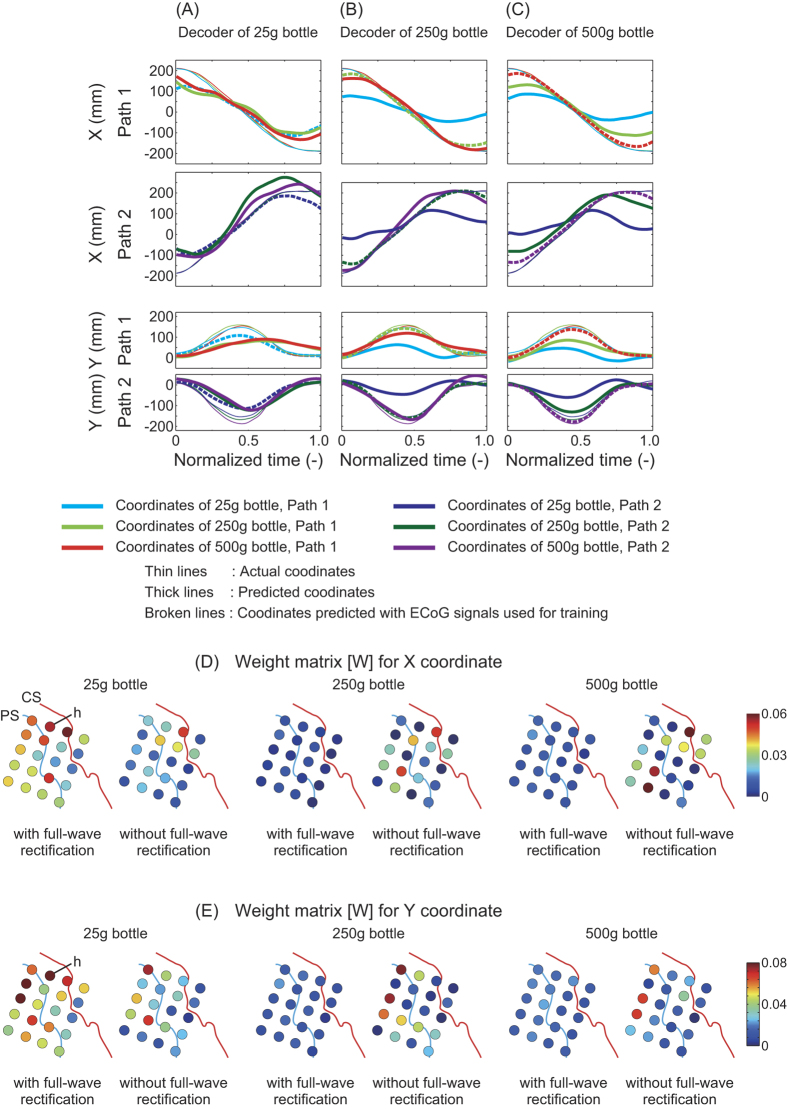
X and Y coordinates predicted with decoders trained with signal data from patient 1 for only one bottle mass. All curves are averaged across all trials in each session. Predictions executed with signals used once in the training phase are denoted with broken lines. (**A**–**C**) Decoders trained with the 25-g, 250-g and 500-g bottles, respectively.

We trained a decoder with complex data involving different masses and opposite trajectories to study its feasibility for neuroprosthetic control. We compared our decoder with a decoder designed for a single mass by applying weight matrices [*W*] trained using a single bottle mass to predict trajectories for all three masses. Figure 7A shows X and Y coordinates predicted using a decoder trained with a data set from patient 1 performing trials with the 25-g bottle (selected due to it having the lowest RMSE). Using the 250-g and 500-g decoders (Fig. 7B,C), prediction for 25 g (cyan/blue lines) showed deficient trajectory compared with actual trajectory. This implies that the weights of the single mass decoder included intrinsic information for the given mass. This mass-specific decoder could not generate trajectory for other conditions. Figure 7D and E show spatial distributions of weight matrices [*W*] used in the three mass-specific decoders. For the 25-g decoder with full-wave rectification, channel *h* showed higher weight values than those of the 250-g and 500-g decoders. Since many intrinsic- and extrinsic-like neurons exist under the area of channel *h*, intrinsic-like neurons may have influenced the training for trajectory decoding.

Predicted biceps brachii activity for patient 1 using one of the decoders obtained with LOO-CV in each session is shown in Supplementary Fig. S6. Although predicted curves slightly differed from each other, they did not fit well with actual muscle activity as compared with the decoders in Fig. 6. Triceps brachii and anterior deltoid activity of patient 1 predicted with mass-specific decoders are also shown in Supplementary Fig. S7.

**Figure 8:**
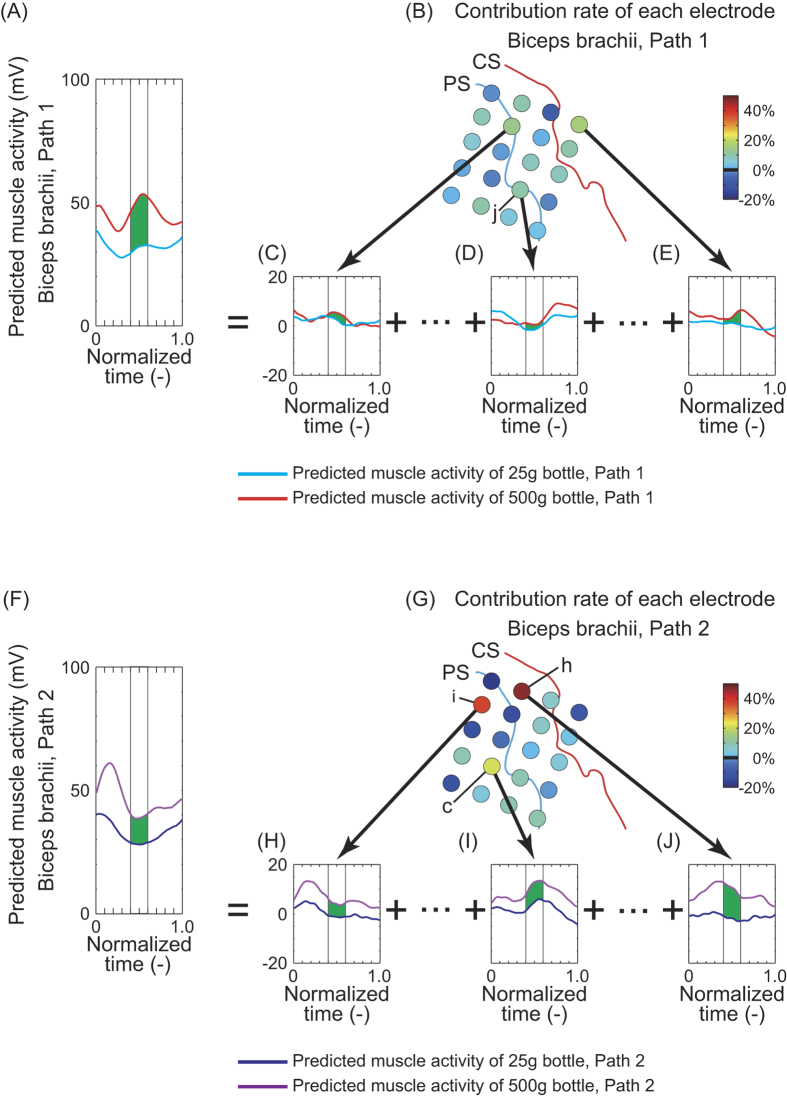
Contribution of each channel to biceps brachii activity prediction for patient 1. (**A**) Muscle activities for the 25-g and 500-g bottles along path 1 were predicted with all ECoG signals (20 channels) using a decoder which showed the best RMSE in LOO-CV. ECoG signals of all bottles and paths were used to train decoders in the LOO-CV. (**C**–**E**) Muscle activities predicted with only one ECoG channel. A common predictor was used in (**C**) through (**E**) and (**A**). Summation of predicted muscle activities (**C**) through (**E**) equals the plot in (**A**). (**B**) The difference between predicted muscle activities for 25 g and 500 g in each channel was averaged from 0.4 T to 0.6 T (green area, where T is a period of each trial), and the percentages of the average difference from (**A**) were expressed as a spatial distribution of contribution to muscle activity prediction. (**F**–**J**) Contribution of each channel to muscle activity prediction for path 2. Channel *h* showed the highest contribution.

We also sought to determine how much channels with high weights contributed to prediction. We predicted biceps brachii activity for patient 1 with one of the decoders obtained after LOO-CV using data for all three masses and two paths to determine the difference in predicted muscle activity for the 25 g and 500 g bottles (Fig. 8A for path 1 and Fig. 8F for path 2). The difference from 0.4 T to 0.6 T was averaged (green area in Fig. 8A and F). To evaluate contribution of each channel, we also predicted muscle activity again using ECoG signals of only one channel and obtained average differences between predicted muscle activities for the 25 g and 250 g bottles at each channel (for example, Fig. 8C–E for path 1, Fig. 8H–J for path 2). Figure 8B and G express percentages of the average difference for each channel compared to those in Fig. 8A and F. Channel *h* showed the highest contribution rate.

We also calculated contribution of each channel to trajectory prediction for the 500 g bottle (Supplementary Fig. S8). Predicted trajectories for paths 1 and 2 were compared (green area). Channels *a* and *b* in M1, and *c, e* and *f* in PM showed high contribution rates.

## Discussion

We used sparse linear regression to predict both bottle trajectory and muscle activity based on ECoG signals. To vary intrinsic parameters for muscle activity while keeping extrinsic parameters unchanged, we asked our patients to carry bottles of different masses along specified paths. Predicted trajectories and muscle activities were evaluated with RMSE and CC. To reduce stress and discomfort, recordings were performed in short durations and in their hospital rooms rather than an electromagnetically shielded room. Their postures were also unstable, because they sat on their beds. In addition, patient 1 had only 20 channels in her electrode array around the sensorimotor cortex (Fig. 1A). However, patient 1 typically showed the best RMSE and CC values (Fig. 4 and Supplementary Fig. S3) among the three patients. Patient 2 was not always able to keep the bottle along the specified path (see left three columns in Supplementary Fig. S1) and changed how she supported it in each session. We surmise that she was learning to carry it using as little energy as possible during the course of the three sessions. This learning likely made our prediction difficult. Although the results of patient 2 showed similar tendencies to those of patient 1, the features were less clear (Supplementary Figs S9 and S10). Patient 3 had the lowest RMSE and CC values among the three patients for trajectory prediction. One possible explanation for this is the trajectory patient 3 applied. She tried to pause at the via-points every trial (Supplementary Fig. S2). This complex movement may have affected decoding accuracy. Another explanation might be the site of patient 3’s brain lesion. Patient 3 had a cerebral congenital lesion near PM and M1, which might have influenced the ECoG. Therefore, results for patient 3 were difficult to interpret (Supplementary Figs S11 and S12).

For our decoders, we used both rectified and unrectified features in the weight selection. We found that using both provided the highest decoding performance (Supplementary Fig. S5). At the least, all rectified frequency bands and unrectified low frequency bands are necessary to obtain satisfactory performance.

Previous studies reported that weight values from δ and γ bands were effective and informative in trajectory decoding^36^,^37^,^38^. Crone *et al*. showed that high γ activity in response to a wide variety of experimental tasks was useful as a general electrophysiological index of cortical processing. Hammer *et al*. showed that the δ and high γ bands were superior to other bands for the prediction of direction, position, velocity, and acceleration. They further proposed the neuronal population activity model and stated that large neuronal population activities such as those of local field potential and ECoG provided better prediction performance than single or multi-unit activity. Pistohl *et al*. reported that the accuracy of grasp-type classification using power spectrums in high (75~170 Hz) and low (<4 or 5 Hz) frequency bands was greater than that using intermediate frequency bands (6~15 Hz and 17~40 Hz). In the current study, weights for the δ, θ and γ bands were higher than those of the other bands. This result is consistent with previous studies decoding for arm movement^7^, finger movement^14^, velocity^37^, and grasp type^38^.

Weight values for the γ bands tended to influence decoding for muscle activity, while those of the unrectified δ band influenced decoding for XY coordinates (Supplementary Fig. S5). In particular, weight values of γ bands from channels *a* and *h* (M1 area) were considerably higher for muscle activity decoding compared with XY-coordinate decoding. This finding matches well with previous studies on high δ contribution to movement^7^,^14^,^37^ and high γ contribution to muscle activity^17^ and object weight^38^.

Since the predicted results were the sum of the product of the frequency features and their weights for both XY-coordinate and muscle activity decoding, components with higher weight values were considered more informative for a specific type of decoding. This is consistent with the conceptual schematic model^36^ and the neuronal population activity model^37^. When neurons in PM and M1 spike for motor planning or motor control, diverse changes in neuronal population activities occur, according to the balance of excitatory and inhibitory synaptic input. These changes may be represented in the frequency bands of local field potential and ECoG. This could be why all frequency bands were needed for effective prediction, and also emphasizes the importance of selecting ECoG electrodes of appropriate size.

The decoder weights trained with each path showed similar patterns for X and Y coordinates (Supplementary Fig. S13). The highest decoder channels *a* and *b* for path 1 were located near each other. Channel *l* decoders for path 2 were located in the same area. However, weights for the decoder trained on all bottle masses and paths showed different distributions (Fig. 5E,F). It was for this reason that the high weights in both paths, such as channel *a*, stood out. Other weights, such as those for channel *k*, were low, as they could not satisfy the demand of the different trajectories. Consequently, the decoder weights trained with all data were more useful to our analysis than the task-specific weights.

Kakei *et al*. indicated that (1) neurons in M1 exhibited intrinsic-like properties, and (2) neurons in both the ventral PM and M1 exhibited extrinsic-like properties^29^. We compared products of temporal frequency features and weights to evaluate whether channels contributed more to XY-coordinate prediction or muscle activity prediction. Our analysis showed results consistent with those of Kakei *et al*. For example, the weight at channel *h* (upper part of M1) was much higher in muscle activity prediction (Fig. 6) than in trajectory prediction (Fig. 5). This could imply that channel *h* included a large amount of EMG information. Figures 8J and G showed that channel *h* gave the highest contribution compared to other channels. Therefore, this channel included the most information about the difference in muscle activity for the 25-g and 500-g bottles along path 2. Channel *a* (right side of channel *h*) had high weights for X coordinates in both paths (Supplementary Fig. S13). This channel also had high weights for muscle activity (Fig. 6E) along path 2, but not along path 1 (Fig. 8). Therefore, the area of channel *a* area may include strong extrinsic-like neurons for trajectory and intrinsic-like neurons for movement.

The spatial distribution of the weight matrix for X-coordinate prediction was not significantly high in PM (Fig. 5E). The differences among channels in contribution for the biceps brachii along path 1 were vague, although a few channels along path 2 had high contribution rates (Fig. 8B and G). Natural hand movement has been shown to be roughly straight with a gentle convex curve^39^,^40^,^41^. Uno *et al*. reported that the speed profile of convex curved paths had only one peak, while those of concave curved path had two peaks. They also reported that horizontal free movements between two targets were always slightly convex curved, because of the complex kinematics and dynamics of the musculoskeletal system. Indeed, the path 1 task was relatively easy for participants, as it involved a natural combination of joint movements, such as internal rotation of the shoulder joint, flexion of the elbow joint and so forth. To carry the bottle along path 2, patients had to draw the object up toward their chest while simultaneously carrying it from their left to right side. This motion is not as common in everyday life and may have forced patients to plan directions and routes before movement onset. These differences for paths 1 and 2 may have influenced the distributions in Fig. 5E and Fig. 8B.

From the viewpoint of neuroprosthetic control, it is ideal that trajectory and muscle activity prediction are based on extrinsic and intrinsic coordinates, respectively. Otherwise, the prosthesis would deviate from the user’s intended course when given an object of different mass, as shown in Fig. 7. When predicting muscle activity from ECoG, inaccuracies are usually inevitable because ECoG signals are summations of activity from a huge number of neurons under each electrode. Accuracy, however, could be improved by training decoders on ECoG recorded during manipulation of objects with various masses.

## Additional Information

**How to cite this article:** Nakanishi, Y. *et al*. Mapping ECoG channel contributions to trajectory and muscle activity prediction in human sensorimotor cortex. *Sci. Rep.* **7**, 45486; doi: 10.1038/srep45486 (2017).

**Publisher's note:** Springer Nature remains neutral with regard to jurisdictional claims in published maps and institutional affiliations.

## Supplementary Material

Supplementary Information
