# Supplementary material for: Mapping ECoG channel contributions to trajectory and muscle activity prediction in human sensorimotor cortex
Source: Sci Rep. 2017 Mar 31;7:45486. doi: 10.1038/srep45486 (PMC5374467; doi:10.1038/srep45486)
Supplement: Supplementary Information [file srep45486-s1.pdf]

Supplementary Information

Mapping ECoG channel contributions to trajectory and muscle activity prediction in human sensorimotor cortex

Yasuhiko Nakanishi[1,¶], Takufumi Yanagisawa[2,3,4,5,¶], Duk Shin[6*],

Hiroyuki Kambara[1], Natsue Yoshimura[1], Masataka Tanaka[3], Ryohei Fukuma[3,4],

Haruhiko Kishima[3], Masayuki Hirata[2,3], Yasuharu Koike[1]

1: Institute of Innovative Research, Tokyo Institute of Technology, Yokohama, Japan

2: Division of Clinical Neuroengineering, Global Center for Medical Engineering and Informatics, Osaka University, Osaka, Japan

3: Department of Neurosurgery, Osaka University Medical School, Osaka, Japan

4: ATR Computational Neuroscience Laboratories, Japan

5: Division of Functional Diagnostic Science, Osaka University Graduate School of Medicine

6: Department of Electronics and Mechatronics, Tokyo Polytechnic University, Atsugi, Japan

*: Corresponding author.

Duk Shin: d.shin@em.t-kougei.ac.jp

1583 Iiyama, Atsugi, Kanagawa 243-0297, Japan

Tel : +81 46 242 4111; Fax : +81 46 242 3000

Supplementary Figure Legends

Fig. S1 Actual trajectories by patient 2 for the 25g, 250g and 500g bottles (three left columns). The right column shows coordinates averaged across trials in each session.

Fig. S2 Actual trajectories by patient 3 for the 25g, 250g and 500g bottles (three left columns). The right column shows coordinates averaged across trials in each session.

Fig. S3 Pearson's correlation coefficient (CC) of predicted compared to actual trajectories (A) and muscle activity (B). In most cases, CC for patient 1 was higher than that of the other patients for both trajectory and muscle activity prediction.

Fig. S4 Triceps brachii and anterior deltoid activity predicted with decoders trained using ECoG signals from all bottle masses and paths for patient 1. (A-C) Predicted muscle activities of each trial for the 25g, 250g and 500g bottles, respectively. (D) Averages across trials for predicted muscle activity (thick lines) compared with actual activity averages (thin lines). (E) Spatial distribution of the weight matrix for the triceps brachii activity predictor. Average weight in M1 was significantly higher than that in PM ($p=1.40\times 10^{-5}$, t-test). (F) Spatial distribution of the weight matrix for the anterior deltoid activity predictor. Average weight in PM was higher than that in M1 ($p=5.26\times 10^{-7}$).

Fig. S5 Weights and ratios of frequency bands used for trajectory and muscle activity prediction in patient 1. (A-B) Frequency distributions of weight matrices for X- and Y-coordinate prediction. Weight for the δ band without full-wave rectification was high in both coordinate predictions. (C) Frequency distributions of the weight matrix for biceps brachii activity

prediction. Weights for the δ and θ bands with full-wave rectification had high values. High frequency bands contributed more to muscle activity prediction than to trajectory prediction.

Fig. S6 Biceps brachii activity for patient 1 predicted using decoders which were each trained on a single bottle mass. (A-C) Decoders trained with the 25g, 250g and 500g bottles, respectively. Predicted muscle activities (thick lines) in each panel showed slight differences. However, the differences were too small to fit predicted muscle activity with actual activity (thin lines). (D) Spatial distributions of weights for the three decoders used in (A), (B) and (C).

Fig. S7 Triceps brachii and anterior deltoid activity for patient 1 predicted using decoders which were each trained on a single bottle mass. (A-C) Decoders trained with the 25g, 250g and 500g bottles, respectively. Predicted muscle activities (thick lines) in each panel showed some differences. However, the differences were too small to fit predicted muscle activity with actual activity (thin lines). (D-E) Spatial distributions of weights for the triceps brachii and anterior deltoid decoders used in (A), (B) and (C).

Fig. S8 Trajectories predicted with one of the decoders obtained after LOO-CV using all data sets of the three bottle masses and two paths for patient 1, and the contribution of each channel. (A) X coordinates for the 500g bottle were predicted with the decoder to calculate the difference between path 1 and path 2 from normalized time 0T to 0.2T (green area), where T is the period of each trial. (C-E) X coordinates predicted with the common decoder using ECoG signals of each channel. (B) Percentages of differences for (C-E) to (A) are expressed as contribution of each channel. Channel a had the highest contribution. (F-G) Contributions of each channel for Y coordinate prediction from 0.4T to 0.6T (green area). Channel c showed the highest contribution.

Fig. S9 X and Y coordinates decoded with ECoG signals of patient 2. The predictors were trained with signals for the three object masses and two paths ("lump together" in Fig. 4A). (A-C) Predicted X and Y coordinates of each trial for the 25g, 250g and 500g bottles, respectively. (D) Averages across all trials for predicted trajectory (thick lines) compared with actual trajectories (thin lines). (E) Spatial distribution of the weight matrix for the X-coordinate predictor. The weight at channel p had the highest value. Average weight in M1 was significantly higher than that in PM ($p=3.55 \times 10^{-33}$, t-test). (F) Weight matrix for the Y-coordinate predictor. Channel q had the highest weight value. Average weights for PM and M1 were not significantly different ($p=0.588$).

Fig. S10 Biceps brachii activity predicted using ECoG signals of patient 2. All signals for the three object masses and two paths were used to train the decoder ("lump together" in Fig. 4B). (A-C) Predicted muscle activity of each trial for the 25g, 250g and 500g bottles, respectively. (D) Averages across trials for predicted muscle activity (thick lines) compared with actual activity averages (thin lines). (E) Spatial distribution of the weight matrix for the biceps brachii activity predictor. The weight at channel r (straddling the central sulcus) had the highest value. Average weights for PM and M1 were not significantly different ($p=0.952$, t-test).

Fig. S11 X and Y coordinates decoded with ECoG signals of patient 3. The predictors were trained with signals for the three object masses and two paths ("lump together" in Fig. 4A). (A-C) Predicted X and Y coordinates of each trial for the 25g, 250g and 500g bottles, respectively. (D) Averages across all trials for predicted trajectory (thick lines) compared with actual trajectories (thin lines). (E) Spatial distribution of the weight matrix for the X-coordinate predictor. Average weight for PM was significantly higher than that for M1 ($p=1.36\times10^{-9}$, t-test). (F) Weight matrix for the Y-coordinate predictor. Average weight for PM was significantly higher than that for M1 ($p=1.33\times10^{-30}$).

Fig. S12 Biceps brachii activity predicted using ECoG signals of patient 3. All signals for the three object masses and two paths were used to train the decoder (“lump together” in Fig. 4B). (A-C) Predicted muscle activity of each trial for the 25g, 250g and 500g bottles, respectively. (D) Averages across trials for predicted muscle activity (thick lines) compared with actual activity averages (thin lines). (E) Spatial distribution of the weight matrix for the biceps brachii activity predictor. Average weight for PM was significantly higher than that for M1 (p=0.00245, t-test).

Fig. S13 X and Y coordinates decoders trained with each path. (A-B) The decoders were trained with path 1 only. (C-D) The decoders were trained with path 2 only. The highest decoder channels *a* and *b* for path 1 were located near each other. Channel l decoders for path 2 were located in the same area. However, weights for the decoder trained on all bottle masses and paths showed different distributions (see Fig. 5E, F).

Table S1 p and F values for an ANOVA comparing trajectories among the three bottle masses

Table S2 p and F values for an ANOVA comparing muscle activities among the three bottle masses

Table S3 p and F values for an ANOVA comparing predicted muscle activity for all trials and patients

Patient 2

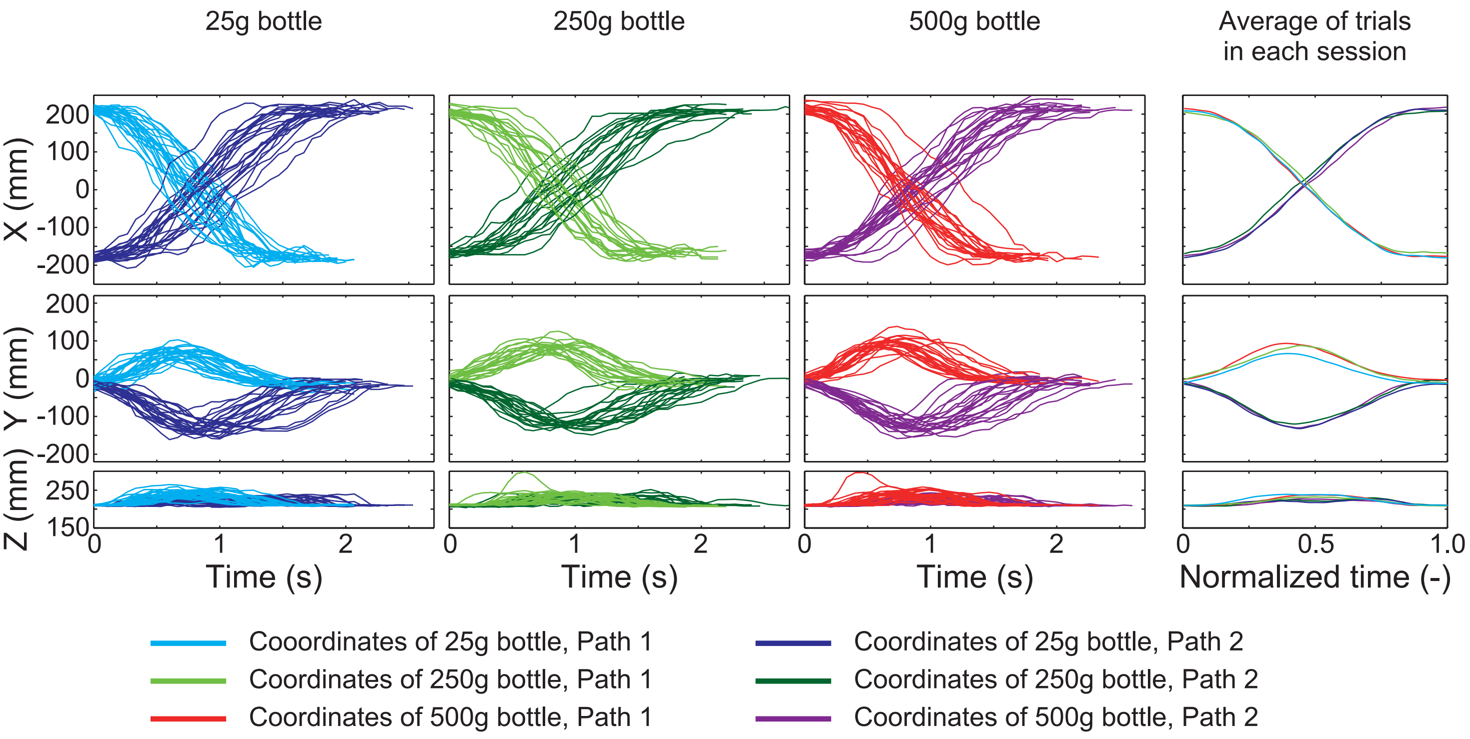

Fig. S1

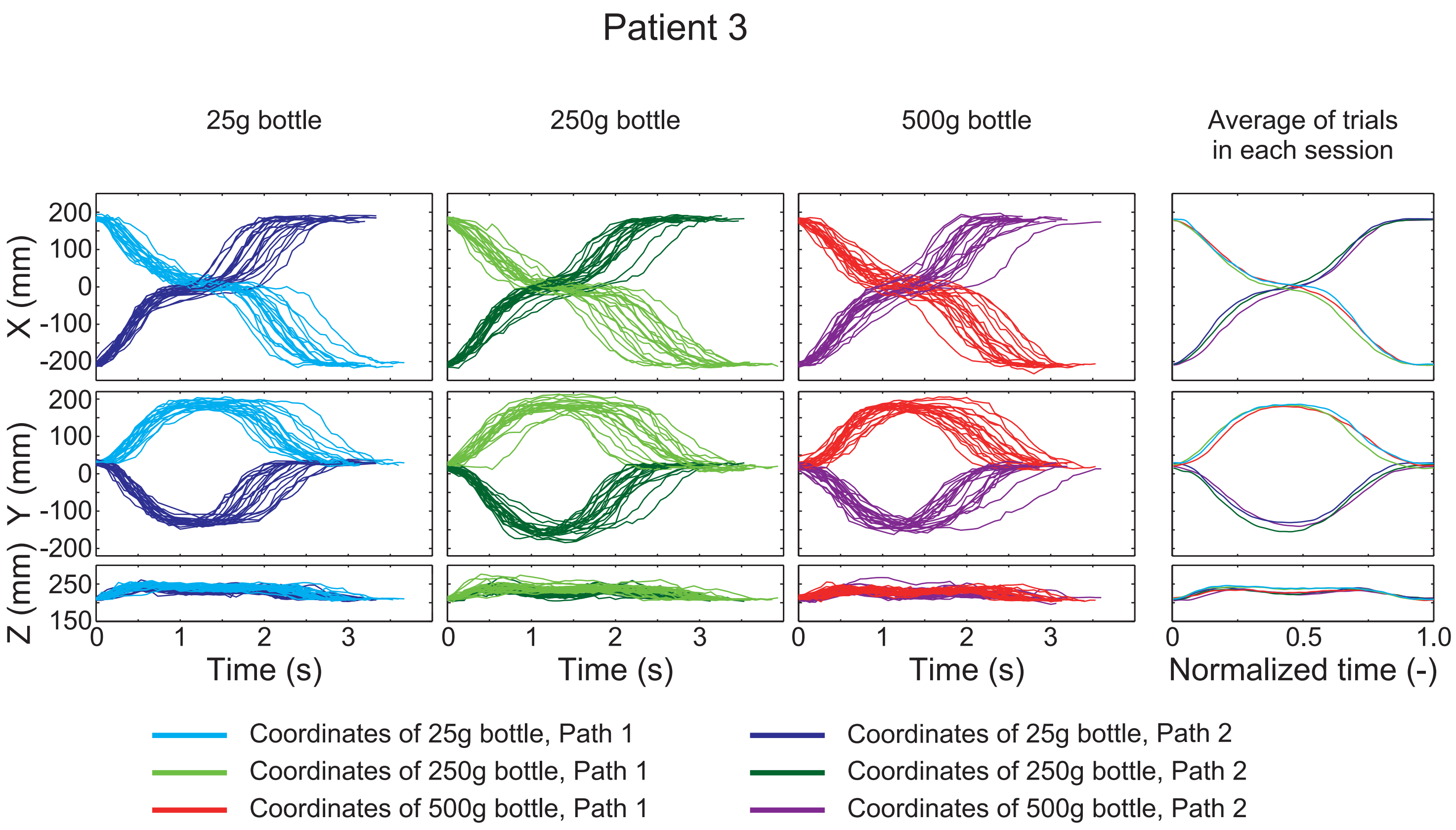
Patient 3
25g bottle
250g bottle
500g bottle
Average of trials in each session
X (mm)
Y (mm)
Z (mm)
200
100
0
-100
-200
250
150
0
1
2
3
0.5
1.0
Time (s)
Normalized time (-)
Coordinates of 25g bottle, Path 1
Coordinates of 250g bottle, Path 1
Coordinates of 500g bottle, Path 1
Coordinates of 25g bottle, Path 2
Coordinates of 250g bottle, Path 2
Coordinates of 500g bottle, Path 2

Fig. S2

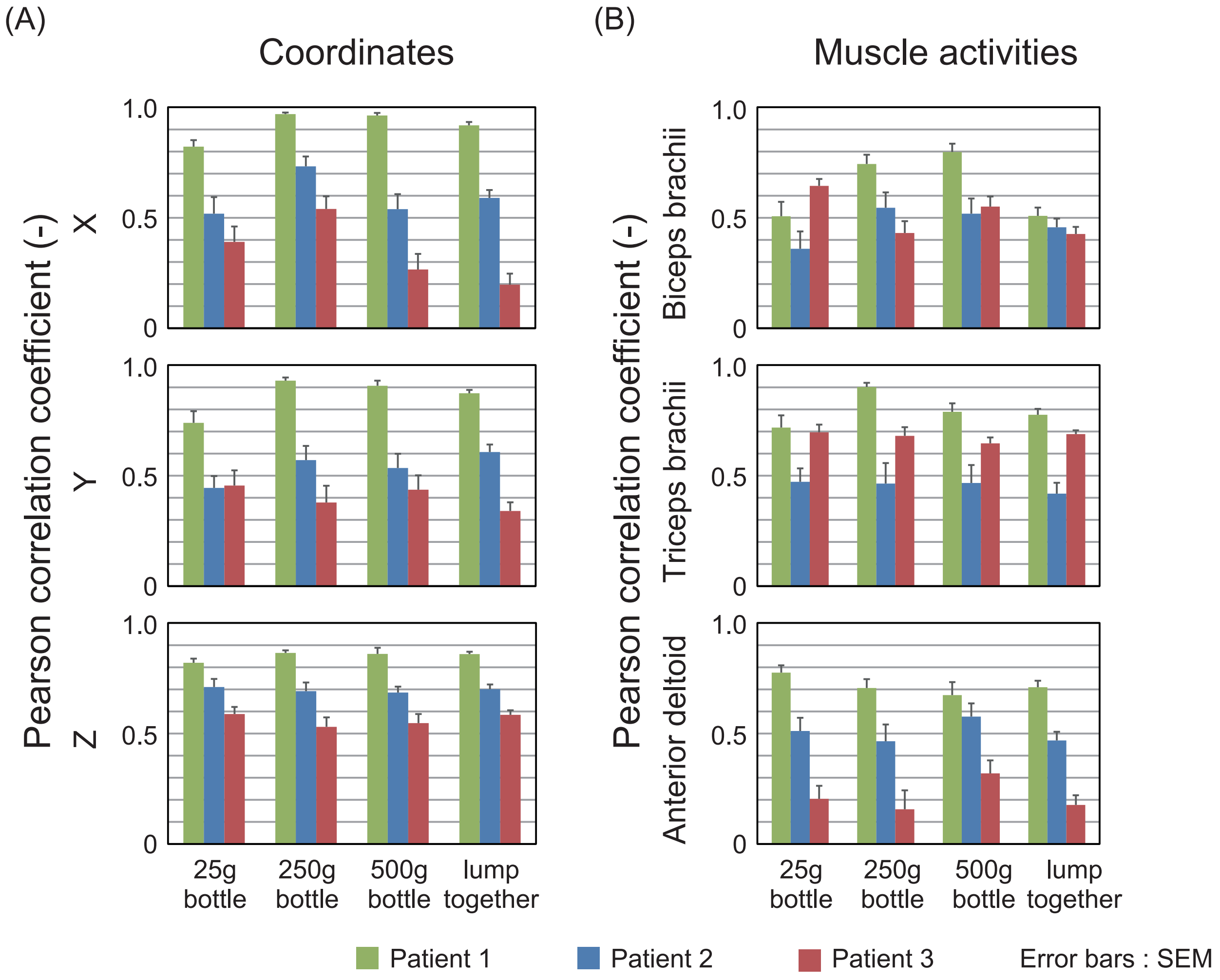
(A)
Coordinates
Pearson correlation coefficient (-)
X
Y
Z
1.0
0.5
0
25g bottle
250g bottle
500g bottle
lump together
(B)
Muscle activities
Pearson correlation coefficient (-)
Biceps brachii
Triceps brachii
Anterior deltoid
Patient 1
Patient 2
Patient 3
Error bars : SEM

Fig. S3

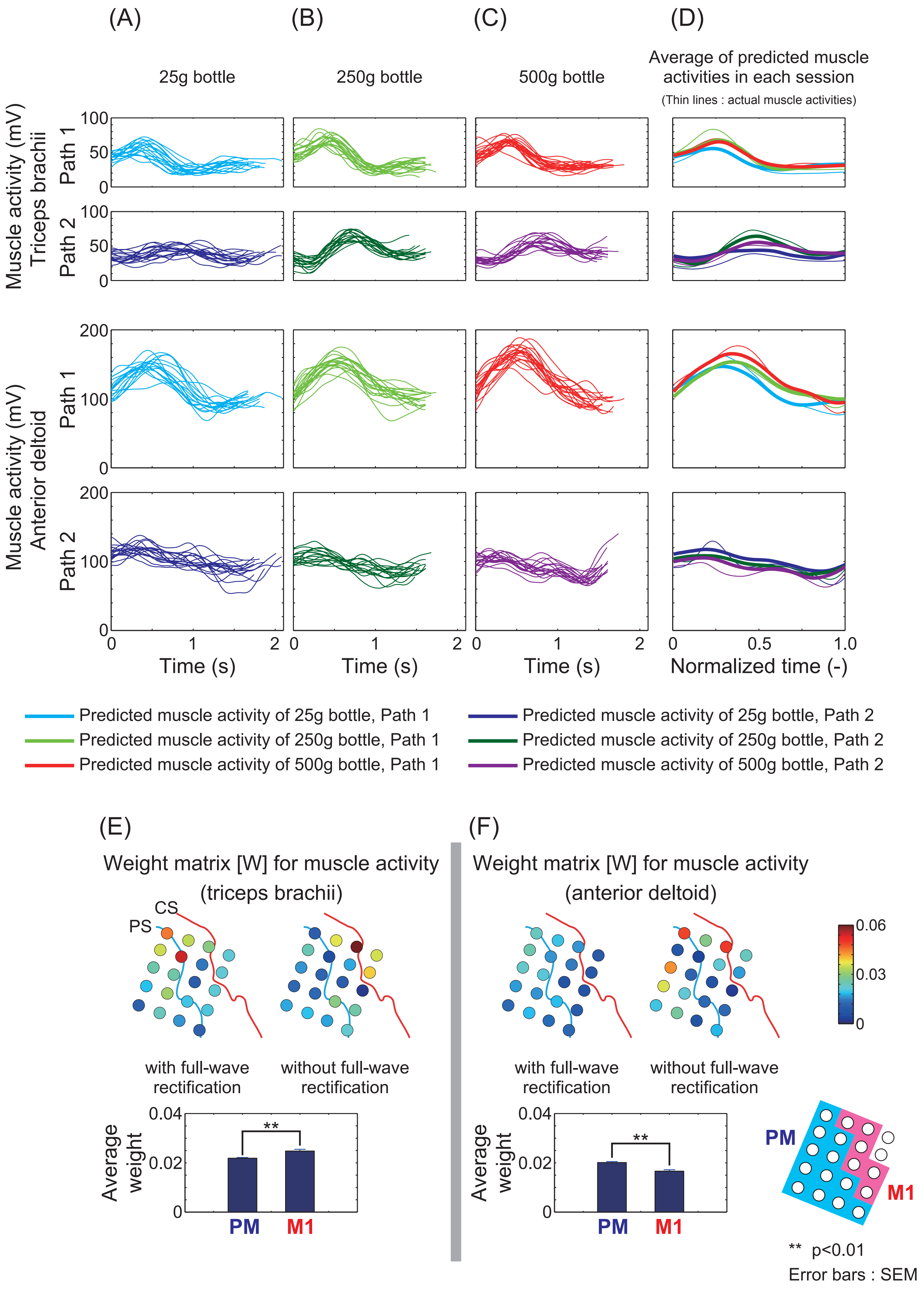

(A)
(B)
(C)
(D)
25g bottle
250g bottle
500g bottle
Average of predicted muscle activities in each session
(Thin lines : actual muscle activities)
Muscle activity (mV) Triceps brachii
Path 1
Path 2
Muscle activity (mV) Anterior deltoid
Path 1
Path 2
Time (s)
Normalized time (-)
Predicted muscle activity of 25g bottle, Path 1
Predicted muscle activity of 250g bottle, Path 1
Predicted muscle activity of 500g bottle, Path 1
Predicted muscle activity of 25g bottle, Path 2
Predicted muscle activity of 250g bottle, Path 2
Predicted muscle activity of 500g bottle, Path 2
(E)
Weight matrix [W] for muscle activity (triceps brachii)
CS
PS
with full-wave rectification
without full-wave rectification
(F)
Weight matrix [W] for muscle activity (anterior deltoid)
with full-wave rectification
without full-wave rectification
Average weight
**
PM
M1
** p<0.01
Error bars : SEM

Fig. S4

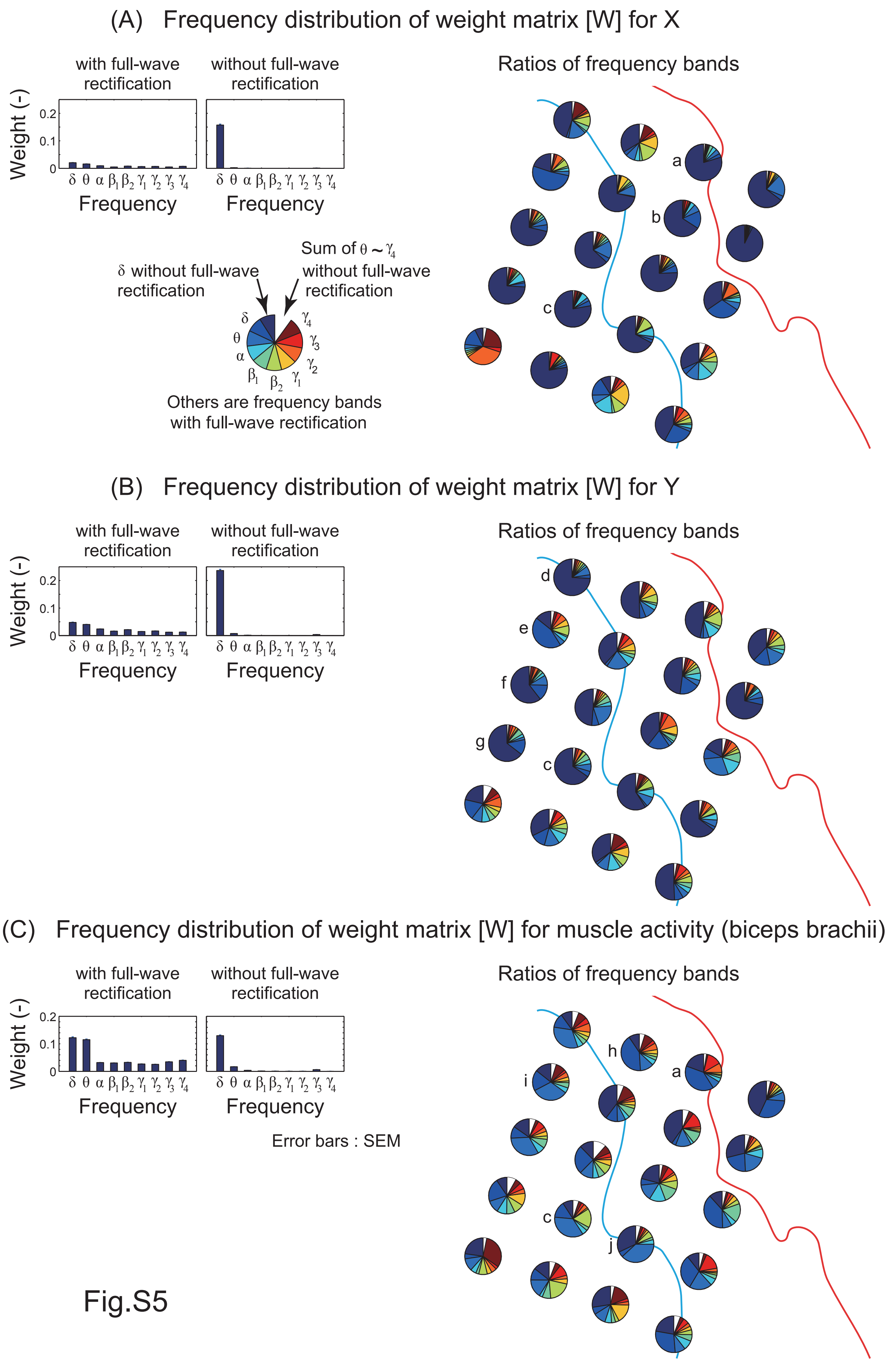

(A) Frequency distribution of weight matrix [W] for X
with full-wave rectification
without full-wave rectification
Weight (-)
Frequency
Ratios of frequency bands
Sum of θ ~ γ4 without full-wave rectification
δ without full-wave rectification
Others are frequency bands with full-wave rectification
(B) Frequency distribution of weight matrix [W] for Y
(C) Frequency distribution of weight matrix [W] for muscle activity (biceps brachii)
Error bars : SEM

Fig.S5

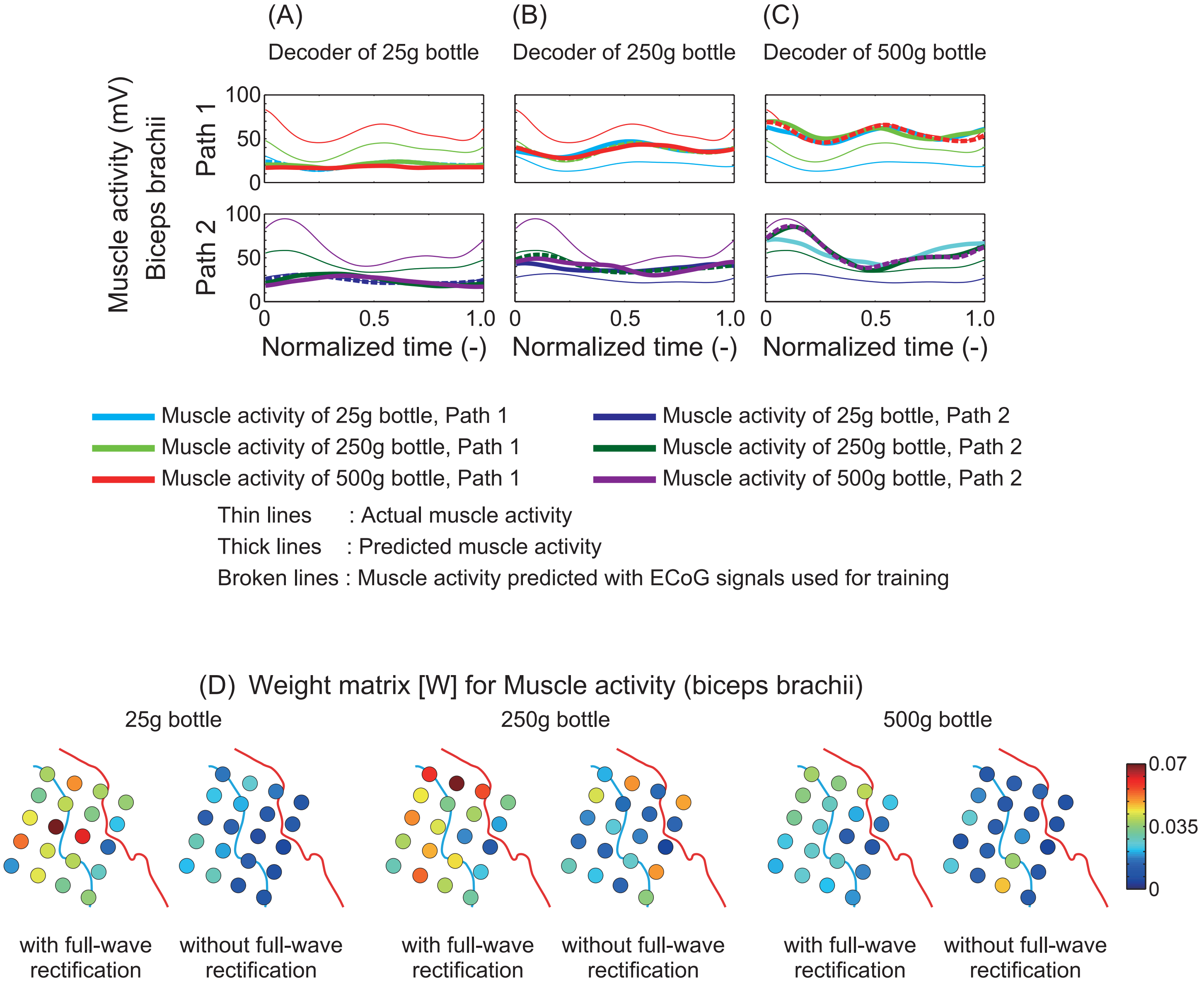
(A)
Decoder of 25g bottle
(B)
Decoder of 250g bottle
(C)
Decoder of 500g bottle
Muscle activity (mV)
Biceps brachii
Path 1
Path 2
100
50
0
0
0.5
1.0
Normalized time (-)
Muscle activity of 25g bottle, Path 1
Muscle activity of 250g bottle, Path 1
Muscle activity of 500g bottle, Path 1
Muscle activity of 25g bottle, Path 2
Muscle activity of 250g bottle, Path 2
Muscle activity of 500g bottle, Path 2
Thin lines : Actual muscle activity
Thick lines : Predicted muscle activity
Broken lines : Muscle activity predicted with ECoG signals used for training
(D) Weight matrix [W] for Muscle activity (biceps brachii)
25g bottle
250g bottle
500g bottle
0.07
0.035
0
with full-wave rectification
without full-wave rectification

Fig. S6

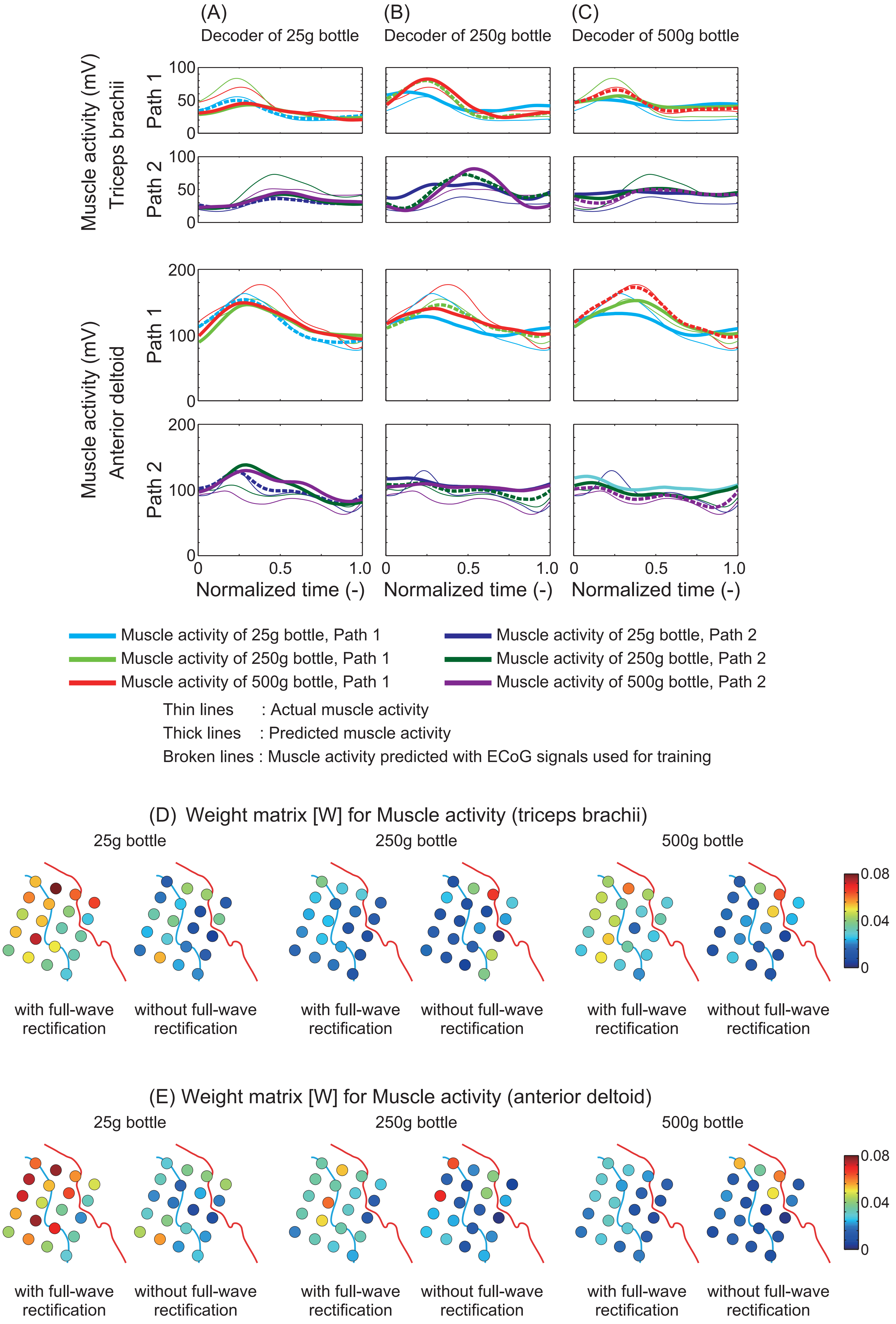
(A)
Decoder of 25g bottle
(B)
Decoder of 250g bottle
(C)
Decoder of 500g bottle
Muscle activity (mV)
Triceps brachii
Path 1
Path 2
100
50
0
Muscle activity (mV)
Anterior deltoid
200
100
0
0.5
1.0
Normalized time (-)
Muscle activity of 25g bottle, Path 1
Muscle activity of 250g bottle, Path 1
Muscle activity of 500g bottle, Path 1
Muscle activity of 25g bottle, Path 2
Muscle activity of 250g bottle, Path 2
Muscle activity of 500g bottle, Path 2
Thin lines : Actual muscle activity
Thick lines : Predicted muscle activity
Broken lines : Muscle activity predicted with ECoG signals used for training
(D) Weight matrix [W] for Muscle activity (triceps brachii)
25g bottle
250g bottle
500g bottle
with full-wave rectification
without full-wave rectification
0.08
0.04
0
(E) Weight matrix [W] for Muscle activity (anterior deltoid)
25g bottle
250g bottle
500g bottle
with full-wave rectification
without full-wave rectification
0.08
0.04
0

Fig. S7

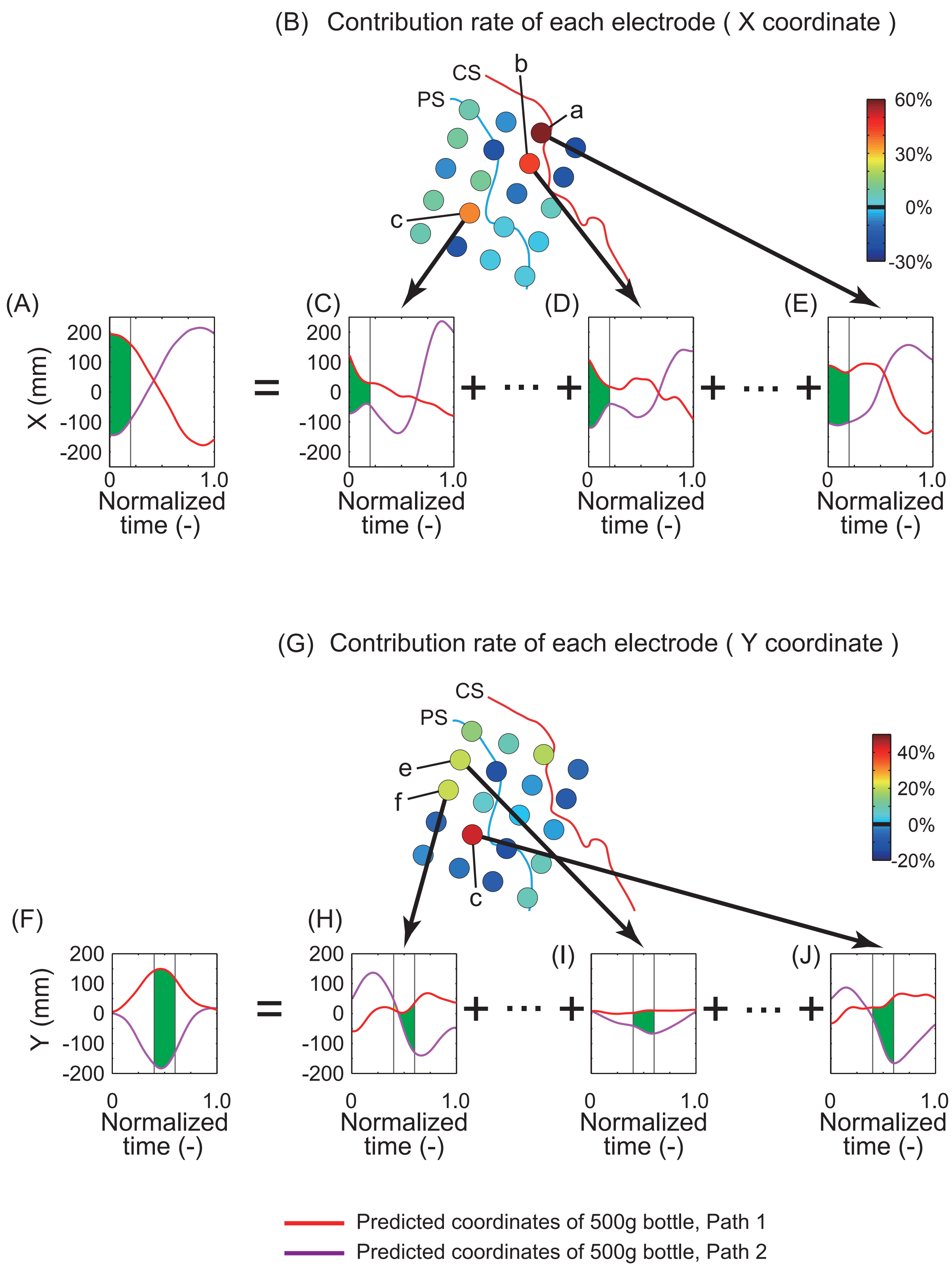
(B) Contribution rate of each electrode (X coordinate)
CS
PS
a
b
c
60%
30%
0%
-30%
(A)
(C)
(D)
(E)
X (mm)
200
100
0
-100
-200
0
1.0
Normalized time (-)
(G) Contribution rate of each electrode (Y coordinate)
CS
PS
e
f
c
40%
20%
0%
-20%
(F)
(H)
(I)
(J)
Y (mm)
Predicted coordinates of 500g bottle, Path 1
Predicted coordinates of 500g bottle, Path 2

Fig. S8

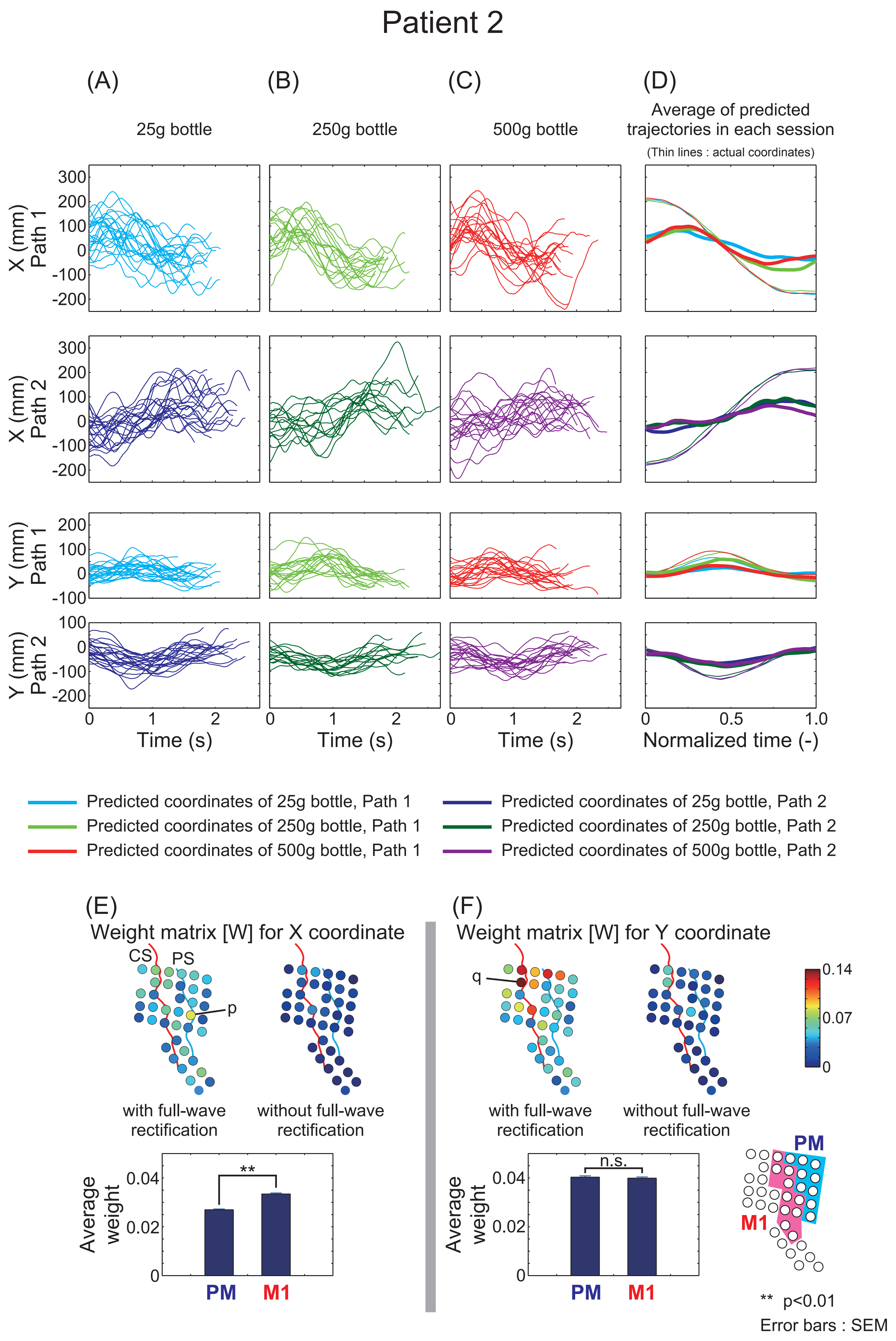

Patient 2
(A)
(B)
(C)
(D)
25g bottle
250g bottle
500g bottle
Average of predicted trajectories in each session
(Thin lines : actual coordinates)
X (mm) Path 1
X (mm) Path 2
Y (mm) Path 1
Y (mm) Path 2
Time (s)
Normalized time (-)
Predicted coordinates of 25g bottle, Path 1
Predicted coordinates of 250g bottle, Path 1
Predicted coordinates of 500g bottle, Path 1
Predicted coordinates of 25g bottle, Path 2
Predicted coordinates of 250g bottle, Path 2
Predicted coordinates of 500g bottle, Path 2
(E)
Weight matrix [W] for X coordinate
CS
PS
p
with full-wave rectification
without full-wave rectification
(F)
Weight matrix [W] for Y coordinate
q
Average weight
PM
M1
**
n.s.
** p<0.01
Error bars : SEM

Fig. S9

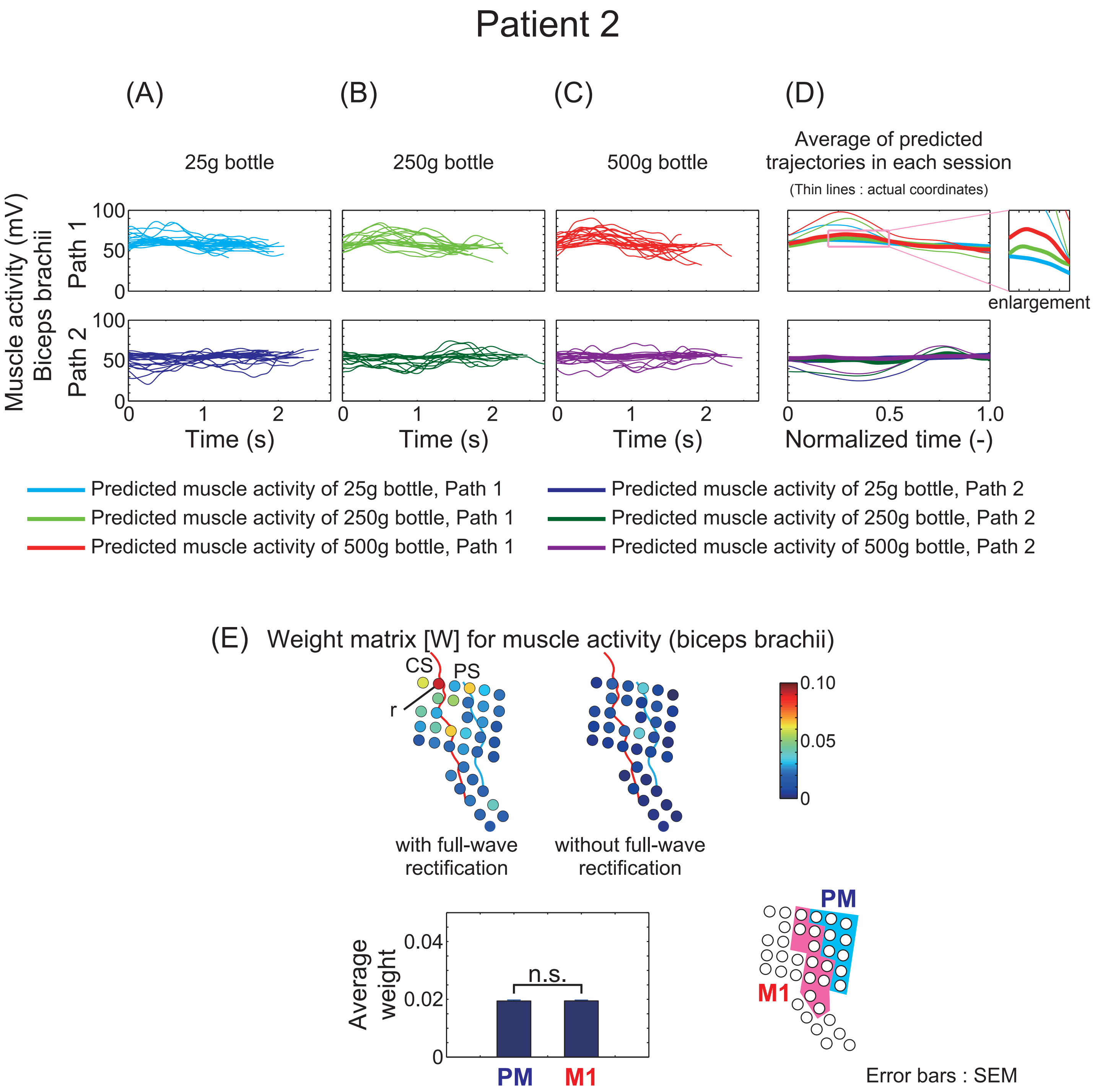

Patient 2
(A)
(B)
(C)
(D)
25g bottle
250g bottle
500g bottle
Average of predicted trajectories in each session
(Thin lines : actual coordinates)
Muscle activity (mV)
Biceps brachii
Path 1
Path 2
100
50
0
0
1
2
Time (s)
0.5
1.0
Normalized time (-)
enlargement
Predicted muscle activity of 25g bottle, Path 1
Predicted muscle activity of 250g bottle, Path 1
Predicted muscle activity of 500g bottle, Path 1
Predicted muscle activity of 25g bottle, Path 2
Predicted muscle activity of 250g bottle, Path 2
Predicted muscle activity of 500g bottle, Path 2
(E) Weight matrix [W] for muscle activity (biceps brachii)
CS
PS
r
with full-wave rectification
without full-wave rectification
0.10
0.05
0
Average weight
0.04
0.02
n.s.
PM
M1
Error bars : SEM

Fig. S10

Patient 3

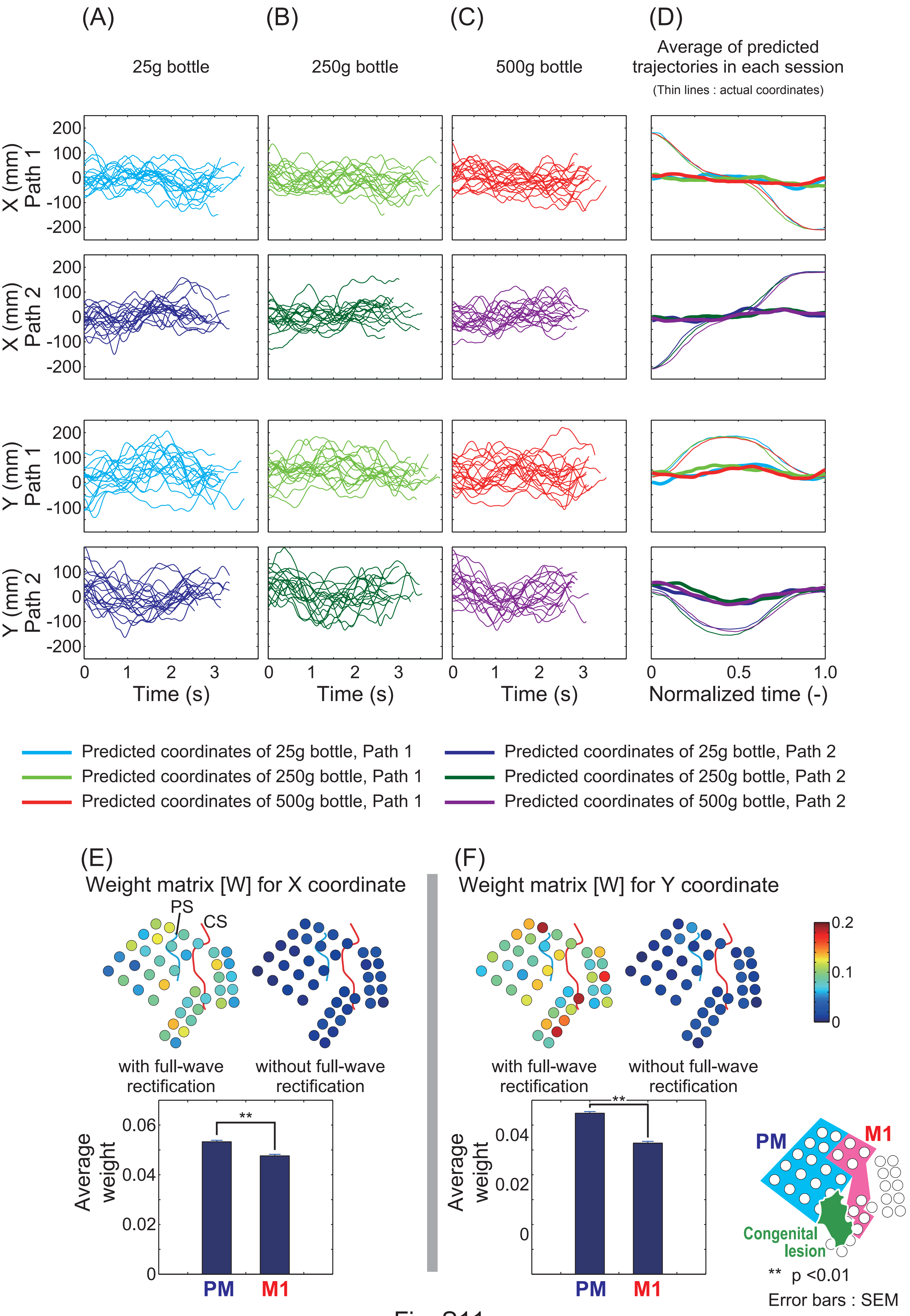

Fig. S11

Patient 3

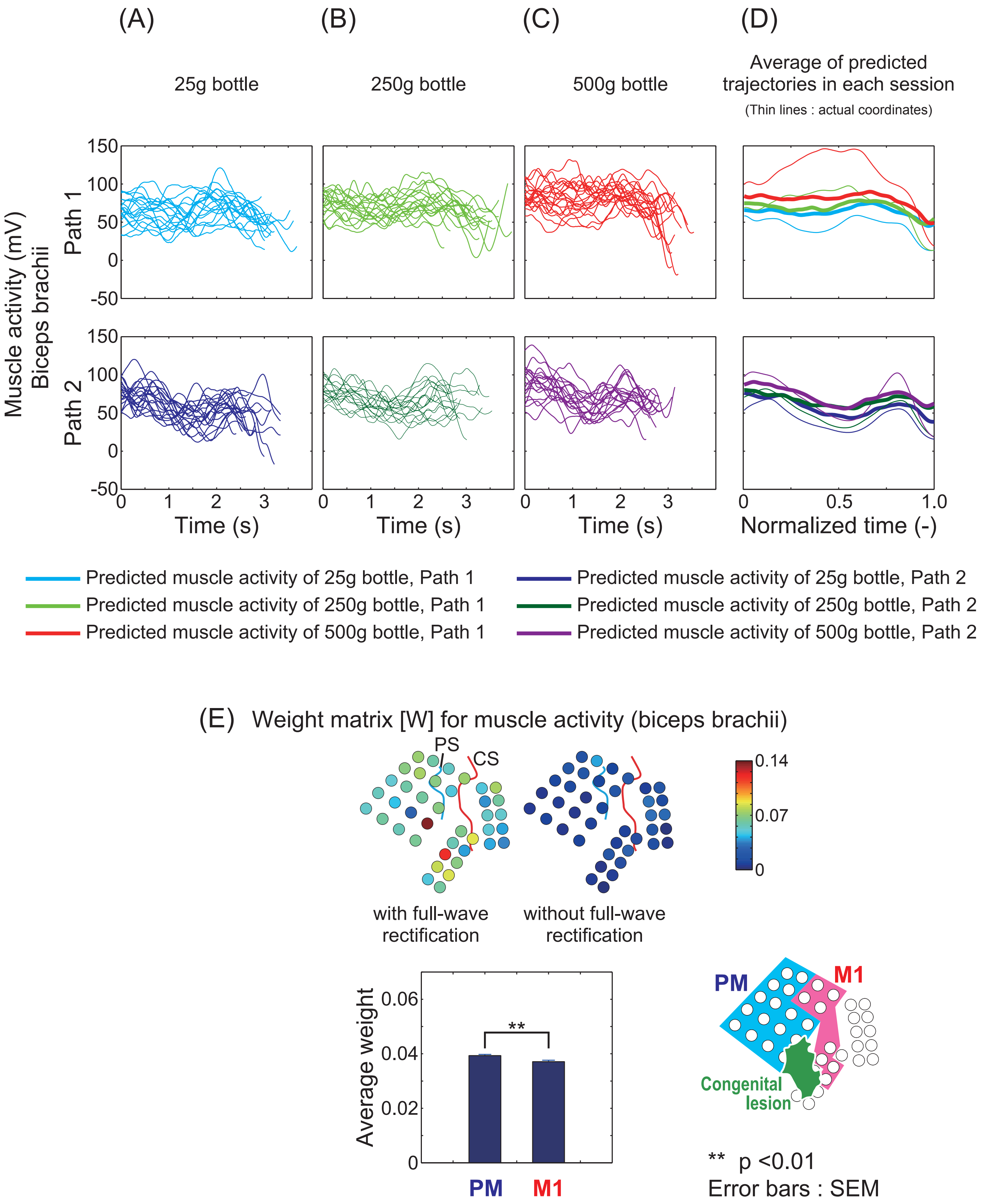

Fig. S12

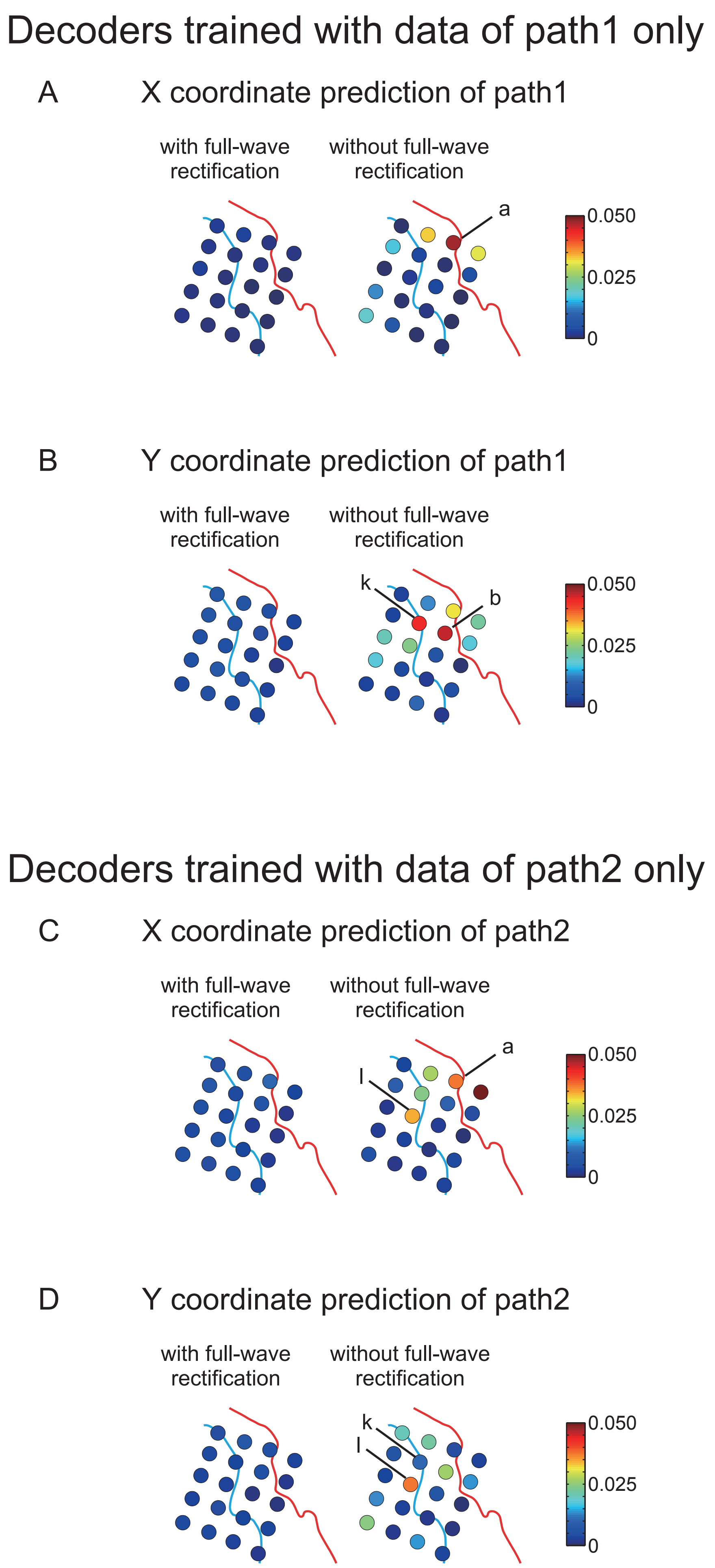
Decoders trained with data of path1 only
A
X coordinate prediction of path1
with full-wave rectification
without full-wave rectification
a
0.050
0.025
0
B
Y coordinate prediction of path1
with full-wave rectification
without full-wave rectification
k
b
0.050
0.025
0
Decoders trained with data of path2 only
C
X coordinate prediction of path2
with full-wave rectification
without full-wave rectification
l
a
0.050
0.025
0
D
Y coordinate prediction of path2
with full-wave rectification
without full-wave rectification
k
l
0.050
0.025
0

Fig.S13

Table S1：p and F values for an ANOVA comparing trajectories among the three bottle masses

X, path 1

		Time (-)		
Patient		0.25 T	0.50 T	0.75 T
#1	p	0.076	0.530	0.302
	F(2,53)	2.709	0.643	1.224
#2	p	0.779	0.773	0.626
	F(2,59)	0.251	0.259	0.473
#3	p	0.219	0.008	0.096
	F(2,53)	1.561	5.325	2.446

Y, path 1

		Time (-)		
Patient		0.25 T	0.50 T	0.75 T
#1	p	0.002	0.043	0.406
	F(2,53)	6.820	3.331	0.916
#2	p	0.001	0.000003	0.028
	F(2,59)	8.209	15.889	3.811
#3	p	0.083	0.084	0.003
	F(2,53)	2.605	2.601	6.496

X, path 2

		Time (-)		
Patient		0.25 T	0.50 T	0.75 T
#1	p	0.736	0.721	0.935
	F(2,47)	0.308	0.330	0.067
#2	p	0.352	0.159	0.031
	F(2,53)	1.064	1.906	3.706
#3	p	0.00002	0.028	0.310
	F(2,51)	13.244	3.847	1.200

Y, path 2

		Time (-)		
Patient		0.25 T	0.50 T	0.75 T
#1	p	0.079	0.0000002	0.559
	F(2,47)	2.684	22.042	0.589
#2	p	0.278	0.069	0.200
	F(2,53)	1.310	2.820	1.658
#3	p	0.0007	0.000002	0.022
	F(2,51)	8.353	17.457	4.129

T: period of each trial

Table S2 : p and F values for an ANOVA comparing muscle activities among the three bottle masses

Biceps brachii
Path 1

		Time (-)		
Patient		0.25 T	0.50 T	0.75 T
#1	p	1.593×10^{-26}	1.213×10^{-20}	1.238×10^{-18}
	F(2,53)	222.815	123.054	99.102
#2	p	5.532×10^{-3}	0.0183	1.100×10^{-4}
	F(2,59)	5.683	4.285	10.680
#3	p	1.163×10^{-17}	2.833×10^{-19}	3.555×10^{-16}
	F(2,53)	88.919	106.288	74.945

Triceps brachii
Path 1

		Time (-)		
Patient		0.25 T	0.50 T	0.75 T
#1	p	1.373×10^{-5}	2.063×10^{-6}	1.794×10^{-22}
	F(2,53)	13.933	16.931	148.831
#2	p	2.314×10^{-4}	2.773×10^{-3}	4.549×10^{-4}
	F(2,59)	9.680	6.517	8.792
#3	p	1.335×10^{-3}	1.329×10^{-11}	1.025×10^{-8}
	F(2,53)	7.519	41.683	26.556

Anterior deltoid
Path 1

		Time (-)		
Patient		0.25 T	0.50 T	0.75 T
#1	p	0.121	4.309×10^{-5}	5.239×10^{-7}
	F(2,53)	2.198	12.224	19.236
#2	p	9.896×10^{-8}	3.753×10^{-12}	2.814×10^{-18}
	F(2,59)	21.464	42.467	86.580
#3	p	0.220	1.059×10^{-3}	0.137
	F(2,53)	1.559	7.818	2.064

Biceps brachii
Path 2

	Time (-)			
Patient		0.25 T	0.50 T	0.75 T
#1	p	2.096×10^{-16}	5.997×10^{-14}	5.646×10^{-19}
	F(2,47)	85.705	62.344	116.972
#2	p	8.403×10^{-5}	0.0182	9.309×10^{-3}
	F(2,53)	11.261	4.328	5.115
#3	p	2.650×10^{-10}	2.995×10^{-15}	3.295×10^{-11}
	F(2,51)	35.048	69.145	40.206

Triceps brachii
Path 2

	Time (-)			
Patient		0.25 T	0.50 T	0.75 T
#1	p	5.190×10^{-9}	1.637×10^{-6}	1.387×10^{-8}
	F(2,47)	29.419	17.927	27.252
#2	p	1.614×10^{-3}	6.947×10^{-3}	0.0121
	F(2,53)	7.276	5.466	4.808
#3	p	1.841×10^{-11}	6.430×10^{-5}	4.636×10^{-11}
	F(2,51)	41.722	11.733	39.332

Anterior deltoid
Path 2

	Time (-)			
Patient		0.25 T	0.50 T	0.75 T
#1	p	1.591×10^{-6}	0.0345	0.0170
	F(2,47)	17.977	3.621	4.450
#2	p	2.278×10^{-10}	8.297×10^{-3}	0.293
	F(2,53)	34.751	5.253	1.256
#3	p	0.175	1.908×10^{-4}	4.345×10^{-3}
	F(2,51)	1.801	10.178	6.062

T: period of each trial

Table S3 : p and F values for an ANOVA comparing predicted muscle activities among the three bottle masses

Biceps brachii Path 1

		Time (-)		
Patient		0.25 T	0.50 T	0.75 T
#1	p	1.595×10^{-4}	6.321×10^{-10}	5.765×10^{-5}
	F(2,53)	10.358	32.437	11.802
#2	p	0.0188	0.425	0.417
	F(2,59)	4.256	0.869	0.888
#3	p	0.00737	0.00668	0.00913
	F(2,53)	5.394	5.513	5.137

Triceps brachii Path 1

		Time (-)		
Patient		0.25 T	0.50 T	0.75 T
#1	p	2.708×10^{-4}	3.788×10^{-4}	0.779
	F(2,53)	9.629	9.175	0.251
#2	p	0.0319	0.582	0.239
	F(2,59)	3.653	0.547	1.468
#3	p	0.0415	0.0450	0.129
	F(2,53)	3.381	3.290	2.129

Anterior deltoid Path 1

		Time (-)		
Patient		0.25 T	0.50 T	0.75 T
#1	p	1.873×10^{-4}	4.509×10^{-6}	1.577×10^{-6}
	F(2,53)	10.136	15.668	17.373
#2	p	2.109×10^{-4}	2.235×10^{-4}	0.0641
	F(2,59)	9.803	9.726	2.878
#3	p	0.323	0.986	0.243
	F(2,53)	1.154	0.0143	1.453

Biceps brachii
Path 2

	Time (-)			
Patient		0.25 T	0.50 T	0.75 T
#1	p	1.489×10^{-11}	6.958×10^{-4}	3.360×10^{-4}
	F(2,47)	44.388	8.521	9.528
#2	p	0.368	0.0309	0.131
	F(2,53)	1.020	3.716	2.115
#3	p	0.0609	0.0559	0.0173
	F(2,51)	2.958	3.054	4.399

Triceps brachii
Path 2

	Time (-)			
Patient		0.25 T	0.50 T	0.75 T
#1	p	0.5816	4.367×10^{-10}	3.681×10^{-4}
	F(2,47)	0.548	35.297	9.400
#2	p	0.284	0.0779	0.267
	F(2,53)	1.291	2.680	1.353
#3	p	0.0218	0.00217	0.226
	F(2,51)	4.126	6.935	1.531

Anterior deltoid
Path 2

	Time (-)			
Patient		0.25 T	0.50 T	0.75 T
#1	p	1.161×10^{-4}	3.464×10^{-4}	0.00614
	F(2,47)	11.056	9.485	5.686
#2	p	0.00237	0.00246	0.0923
	F(2,53)	6.791	6.744	2.493
#3	p	0.691	0.209	0.979
	F(2,51)	0.373	1.612	0.0212

T: period of each trial
